# Toward Faster Adsorbent Screening via the Multisite-Whittaker Approximation

**DOI:** 10.1021/acs.langmuir.5c01081

**Published:** 2025-08-05

**Authors:** L. Scott Blankenship, Paul Iacomi

**Affiliations:** † School of Chemistry, University Park, 6123 University of Nottingham, Nottingham NG7 2RD, U.K.; ‡ Surface Measurement Systems, Unit 5, Wharfside, Rosemont Road, London HA0 4PE, U.K.

## Abstract

Measurement of adsorption isotherms is a time-consuming process, which is required for screening adsorbents for various applications. We present a simple method for accurately predicting isotherms for temperatures within 50 K of a single measured isotherm, indeed making it possible to determine an entire adsorption surface, that is, loading as a function of both temperature and pressure. The methodology is an expansion of the Tóth potential approach applied by Whittaker [Whittaker, P. B.; Wang, X.; Regenauer-Lieb, K.; and Chua, H. T. Predicting isosteric heats for gas adsorption. *Physical Chemistry Chemical Physics* **2013**, *15*, 473–482.] The implementation of such a theory has been deployed in the open-source adsorption processing software pyGAPS in order to improve reproducibility.

## Graphical abstract

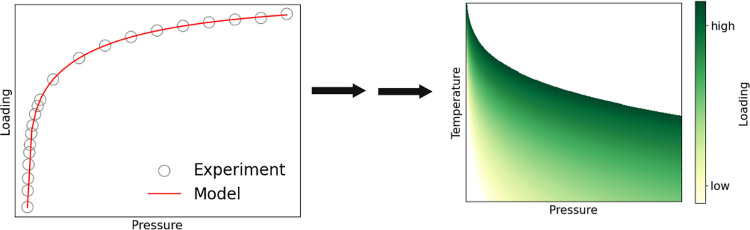

## Introduction

The measurement of adsorption isotherms has become a standard, highly automated process with the advent of off-the-shelf sorption instruments adapted to a variety of purposes. Simultaneously, adsorption data are in high demand both for the physical characterization of porous and finely divided materials and for the determination of the applicability of various sorbents for gas storage or capture applications. The demand for materials exhibiting high capacity and/or selectivity for some molecule of interest is extremely high and leads to rather frequent claims of the next physical or hypothetical “record-breaking” sorbent for some planet-saving application.^1–5^ It is clear that robust screening of adsorbents is necessary, and batteries of experimental tests have been suggested by a variety of researchers, in particular, for direct air carbon capture (DAC) materials.^6–8^ The number of potential adsorbents can be reduced via computational screening,^9–15^ as well as some proposed high-throughput experimental methods;^16–19^ however, robust sorbent screening requires the measurement of several isotherms per adsorbate, per adsorbent. This is not only due to the need for understanding of the temperature dependence of the adsorption/separation process but also results from the need to understand the thermodynamics of the adsorption processes.

Perhaps, the most thorough and robust proposed adsorbent screening process has been proposed by Low et al. in 2023, for DAC materials.^6^ The screening process consists of the measurement of a total of 15 isotherms; seven for CO_2_; three each for N_2_ and H_2_O; and one each for Ar and O_2_, besides a number of other structural and morphological investigations. In the case of CO_2_ isotherms, the 36 h sample preparation was repeated for every temperature, presumably to exclude the possibility of any persistent chemically adsorbed CO_2_ impacting later measurements. As a result, recording the CO_2_ isotherms alone requires at minimum 14 days to complete, if it is generously assumed that the measurement of the isotherms themselves take no more than 12 h. Although every bit of this analysis is essential, Low et al.’s screening procedure can be impractical if a large library of samples is to be considered.

The Clausius–Clapeyron equation^20,21^ (eq 1) allows the isosteric enthalpy of adsorption, Δ*h*_ads_ to be calculated from multiple isotherms measured at appropriately spaced temperatures *T*, using pressures *P* where loadings *n* are identical.

1 $${\frac{\partial \ln P}{\partial T}|}_{n}=-\frac{\Delta h_{ads}}{RT^{2}}$$

This is the approach that most researchers, including Low et al., use to determine Δ*h*_ads_, in lieu of the more direct but also more expensive calorimetric measurements.^6^ Apart from requiring the measurement of multiple isotherms, this method also incurs large uncertainties due to the spacing of the temperatures used. Alternatively, Whittaker et al.^22^ in 2013 reported a method for approximating Δ*h*_ads_ from a single isotherm by combining the Tóth adsorption potential^23,24^ and the fitting parameters from Langmuir or Tóth model isotherms.^22^ This proved to match calorimetric data well (generally within ±15%) for hydrocarbons as well as SF_6_, CO_2_, and N_2_ on silica and zeolites. Subsequently, this method has been used in several publications.^25–29^ The greater potential for the Whittaker approximation is that if its assumptions reflect the underlying adsorption mechanism, then isotherms at other temperatures could conceivably be predicted from a single isotherm. This has actually been demonstrated in a recent publication wherein this method was successfully used to predict hydrogen isotherms at −123 °C from isotherms measured at −196 °C,^30^ thereby allowing a temperature–pressure swing adsorption working capacity to be determined for the activated carbons in question. Indeed, an adsorption surface, i.e., loading as a function of both pressure and temperature, *n*(*p*, *T*), could be calculated.

This work proposes an expansion to Whittaker’s method by allowing the theory to account for multiple adsorption sites. This allows for better fitting of isotherms from a broader range of adsorbent–adsorbate systems and physical conditions and therefore the prediction of adsorption behavior. It is hoped that this will ultimately lead to a faster screening of adsorbents.

## Materials and Methods

### Theory

The Tóth adsorption potential,^23,24^ ε_ads_, is defined by eq 2, where *P*, *P*_sat_, *T*, and *R* are pressure, saturation pressure, temperature, and the molar gas constant, respectively. Ψ is the correction factor to the original Polanyi potential,^31,32^ which is expressed as a differential function of loading, *n* and pressure as shown in eq 3. Equation 3 can be related to the Gibbs spreading pressure when considering monolayer coverage.

2 $$\varepsilon_{ads}\left(P\right)=RT\ln\left(\frac{\Psi P_{sat}}{P}\right)$$

3 $$\Psi ={\frac{n}{P}\frac{dP}{dn}|}_{T}-1$$

Several authors have shown that Δ*h*_ads_ ^33^ can be approximated from the pressure-dependent enthalpy of vaporization, Δ*H*_vap_(*P*), and the adsorption potential, ε_ads_ (eq 4).^34–36^

4 $$-\Delta h_{ads}\approx \varepsilon_{ads}+\Delta H_{vap}\left(P\right)+Z\left(T,P\right)\cdot RT$$

The compression factor, *Z*(*T*, *P*), has little effect on the calculation at low pressure, as the adsorbate can be considered as behaving ideally. As the remaining parameters in eq 4 are precisely known, the difficulty in determining Δ*h*_ads_ comes down to approximating ε_ads_; in effect determining an expression for Ψ in eq 2. Whittaker’s contribution was to state that ε_ads_ can be calculated from parameters from the Tóth or Langmuir unary adsorption isotherm model, eq 5, in terms of the fractional coverage, θ, as shown in eq 7 ^22,37^ via an expression in eq 6. Namely, the affinity constant, *K*, and the system heterogeneity parameter, *t*, come together to approximate the adsorption potential as a function of fractional coverage in eq 7.

5 $$n\left(P\right)=n_{m}\frac{KP}{\sqrt[{t}]{1+{\left(KP\right)}^{t}}}$$

6 $$\Psi ={\left(KP\right)}^{t}$$

7 $$\varepsilon_{ads}\left(\theta \right)=RT\ln\left(KP_{sat}{\left[\frac{\theta^{t}}{1-\theta^{t}}\right]}^{t-1/t}\right)$$

In the case of an isotherm measured at some temperature, *T* above the critical temperature of the adsorbate, an empirical pseudosaturation pressure^38–41^ can be substituted for *P*_sat_ derived from the critical pressure and temperature, i.e., $P_{sat}=P_{c}{\left(T/T_{c}\right)}^{2}$ . As the Tóth model reduces to the Langmuir^42^ equation for *t* = 1, eq 7 can also be applied for Langmuir isotherms, wherein $\varepsilon_{ads}=RT\ln\left(KP_{sat}\right)$ , i.e., the potential is constant as a function of *P* or θ.^22^

Soon after Langmuir proposed his eponymous model for isothermal adsorption, he expanded it to allow for adsorption onto *i* sites where *i* is a positive integer, with each site *i* having its own *K*_*i*_ and *n*_m_*i*__.^43^ This allows for adsorbents that have regions with different affinities for the adsorbate, typically *i* ≤ 3. Pressure-dependent loading is then determined as the linear combination of all sites, as in eq 8. This multisite approach can equally be applied to the Tóth model, with the addition of *t*_*i*_ parameters as in eq 9. The multisite Tóth model been used sparsely—principally for polar, asymmetric adsorbates such as CO_2_ and H_2_O,^44,45^ it should be noted that the utility of this approach is questionable as both *t*_*i*_ and the use of multiple sites purport to account for surface heterogeneity.

8 $$n\left(P\right)=\sum_{i}n_{m_{i}}\frac{K_{i}P}{1+\left(K_{i}P\right)}$$

9 $$n\left(P\right)=\sum_{i}n_{m_{i}}\frac{K_{i}P}{\sqrt[{t_{i}}]{1+{\left(K_{i}P\right)}^{t_{i}}}}$$

Without weighting or proper consideration of the expected properties of each of the adsorption sites, eq 9 can yield parameters of unrealistic or impractical magnitude. A model was developed by Serna-Guerrero and colleagues to describe adsorbate–adsorbent systems, wherein both chemical and physical adsorption processes are important. This is achieved by the linear combination of a Tóth and Langmuir model for the physical and chemical adsorption processes, respectively, as shown in eq 10.^46^ This has been successfully used to model CO_2_ adsorption on amine-functionalized sorbents.^46,47^

10 $$n\left(P\right)={\left[n_{m_{p}}\frac{K_{p}P}{\sqrt[{t_{p}}]{1+{\left(K_{p}P\right)}^{t_{p}}}}\right]}_{physical}+{\left[n_{m_{c}}\frac{K_{c}P}{1+K_{c}}\right]}_{chemical}$$

Equation 10 requires the experimental determination of the relative contribution of the two adsorption sites. In practice, it is not simply eq 9 with *i* = 2 and *t*_2_ = 1, as it requires some coefficient on one of the sites to account for the relative influence of the two sites on the adsorption behavior. Recently, Low et al. proposed that the contribution of chemisorption could be considered to be controlled by Arrhenius kinetics. As shown in eq 11, the chemical portion has the coefficient γ which limits the effect of chemisorption at low temperatures and is governed by the activation energy *E*_*a*,*c*→*p*_ as shown in eq 12.^48^ This equation has been successfully used on a number of systems, wherein the adsorbent contains some discrete chemically reactive or polar sites, and the adsorbate is polar or reactive.^6^ The multisite Tóth model (eq 9) can be expanded to allow the possibility to include this information, resulting in eq 13.

11 $$n\left(P\right)={\left[n_{m_{p}}\frac{K_{p}P}{\sqrt[{t_{p}}]{1+{\left(K_{p}P\right)}^{t_{p}}}}\right]}_{physical}+\gamma \cdot {\left[n_{m_{c}}\frac{K_{c}P}{1+K_{c}P}\right]}_{chemical}$$

12 $$\gamma =\exp\left(-\frac{E_{a,c\to p}}{RT}\right)$$

13 $$n\left(P\right)=\sum_{i}\gamma_{i}n_{m_{i}}\frac{K_{i}P}{\sqrt[{t_{i}}]{1+{\left(K_{i}P\right)}^{t_{i}}}}$$

An expression for Ψ is shown in eq 14 which includes multiple sites as well as the γ parameter present in the ChemiPhysisorption model.

14 $$\Psi =\left[\frac{\sum_{i}\frac{\gamma_{i}n_{m_{i}}K_{i}}{{\left(1+{\left(K_{i}P\right)}^{t_{i}}\right)}^{1/t_{i}}}}{\sum_{i}\frac{\gamma_{i}n_{m_{i}}K_{i}}{{\left(1+{\left(K_{i}P\right)}^{t_{i}}\right)}^{t_{i}+1/t_{i}}}}\right]-1$$

This can be rewritten in terms of θ like Whittaker et al.; however, it does not make the expression any less unwieldy. Further, unlike eq 6, this expression does not easily simplify when used to calculate for ε_ads_ if *i* > 1. However, it does reduce down to eq 6 for *i* = 1 and γ = 1, i.e., single-site Tóth. In the case of all *t*_*i*_ = 1, and all γ_*i*_ = 1—i.e., a multisite Langmuir isotherm, the expression becomes eq 15.

15 $$\Psi =\left[\frac{\sum_{i}\frac{n_{m_{i}}K_{i}}{1+K_{i}P}}{\sum_{i}\frac{n_{m_{i}}K_{i}}{{\left(1+K_{i}P\right)}^{2}}}\right]-1$$

The approximation for heat of adsorption from any multisite Tóth-like isotherm then becomes (eq 16)­

16 $$-\Delta h_{ads}\left(P\right)\approx RT\;\ln\frac{P_{sat}}{P}\left(\frac{\sum_{i}\frac{\gamma_{i}n_{m_{i}}K_{i}}{{\left(1+{\left(K_{i}P\right)}^{t_{i}}\right)}^{1/t}}}{\sum_{i}\frac{\gamma_{i}n_{m_{i}}K_{i}}{{\left(1+{\left(K_{i}P\right)}^{t_{i}}\right)}^{t+1/t}}}-1\right)+\Delta H_{vap}\left(P\right)+Z\left(T,P\right)\cdot RT$$

Isotherms can then be predicted at a given temperature, *T*_p_, from the rearrangement of the Clausius–Clapeyron equation given in eq 17. That is, for some loading, *n* measured at temperature, *T*_e_, and pressure, *P*_e_, for which −Δ*h*_ads_ has been calculated, the pressure *P*_p_ associated with this same *n* can be predicted at a different temperature *T*_p_.

17 $${\ln P_{p}=\frac{-\Delta h_{ads}\left(T_{p}-T_{e}\right)}{RT_{e}T_{p}}+\ln P_{e}|}_{n}$$

Thus, by (i) modeling an experimental isotherm using eq 13, (ii) calculating Δ*h*_ads_ using eq 16, (iii) pressures can be calculated as a function of loading at another temperature *T*_p_. In other words, a predicted isotherm is calculable. In this method, the predicted isotherm can be determined only for loadings in the range of the original isotherm.

It should be noted that this method does not yield the temperature extension parameters. That is, it does not directly yield a fully temperature-dependent adsorption relationship. This may be achievable at a later date.

A table comparing the applicability of all of the models is available in the Supporting Information as S1.

### Software Design

In order to make the implementation of the theory described above both accessible and reproducible, pyGAPS has been extended. All features described herein are available from v4.6.1 and above. Detailed information on pyGAPS can be found in the original publication,^49^ or in the documentation.^50^ Internally, pyGAPS stores sorption data in abstract Isotherm objects, which can either represent data points (PointIsotherm) or express mathematical sorption models (ModelIsotherm). These Isotherm objects can be exported in various formats including AIF,^51^ plotted or further used to obtain such as surface area, pore size distribution, enthalpy of sorption, model fitting, or predict multicomponent sorption using Ideal Adsorbed Solution Theory (IAST). The framework includes a databases of adsorbate properties and equations of state based on the REFPROP^52^ or Coolprop libraries.^53^

In order to implement the expanded Whittaker theory, new isotherm models have been added to pyGAPS, namely, the Dual Site Tóth model (DSToth, eq 9 where *i* = 2) and the ChemiPhysisorption model (ChemiPhysisorption, eq 11). Additional functions have been added to the Adsorbate class, and a new module has been created to calculate the isosteric enthalpies of adsorption using the Whittaker approximation. Finally, a new submodule for predicting isotherms from the enthalpies of adsorption has been created in the newly created prediction module.

A more detailed description of the modifications made to pyGAPS can be found in the Supporting Information from page S5.

## Results and Discussion

This section relies principally on the unary CO_2_ and N_2_ isotherms graciously provided by Low et al. in the AIF format,^51^ measured on the commercial styrene-divinylbenzene sorbents Purolite A110 and Lewatit VP OC 1065, herein referred to as Purolite and Lewatit.^6^ All of the isotherms are displayed in Figure 1a–d. Purolite and Lewatit have roughly similar adsorption behavior for all adsorbates. Calculation of Δ*h*_ads_ and predicting isotherms using the CO_2_ and N_2_ isotherms is discussed in two separate sections below. The results and method for Purolite and Lewatit are similar; therefore, we chose to center the discussion and results on Purolite. Figures S1, S3, and S5–S9 show the results for Lewatit.

**Figure 1:**
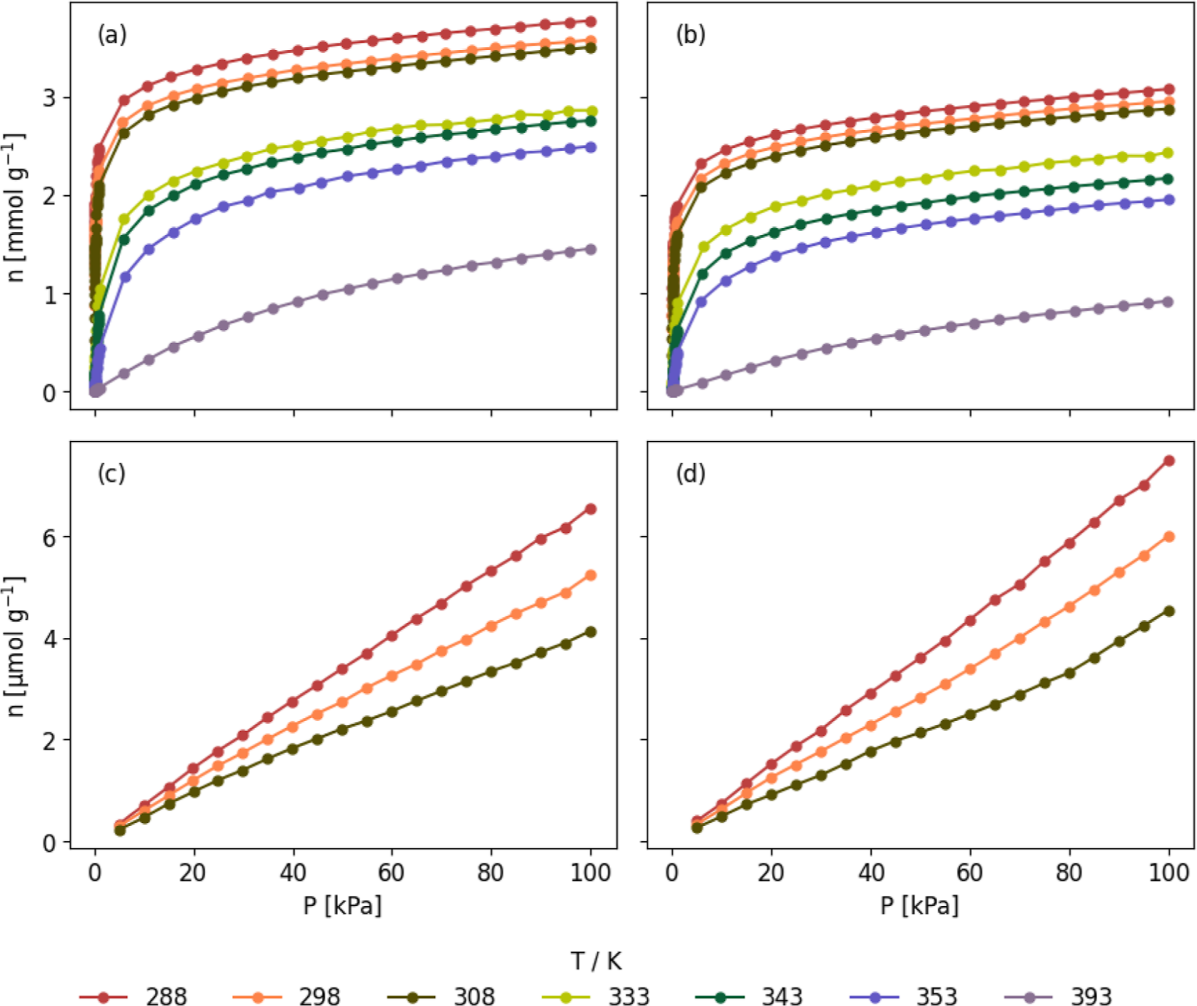
Unary CO_2_ (a, b), N_2_, (c, d), isotherms measured on Purolite (a, c), and Lewatit (b, d) data from Low et al.^6^

### Case Study Using Carbon Dioxide Isotherms

Before the Whittaker approximation is attempted, it is important to examine how the set of Whittaker-consistent model isotherms fits the experimental isotherms. Figure 2a–c displays the dependence of the root-mean-square error (RMSE) of the six Whittaker-consistent models on isotherm temperature for CO_2_ adsorption as well as an example of the fits. The fitting parameters for all models, temperatures, and both adsorbents are shown in Tables S2–S15. For both adsorbents, using a model with more terms reduces the RMSE for all temperatures and as such, dual-site Tóth (DSTóth), triple-site Langmuir (TSLangmuir), and ChemiPhysisorption models have the best fits; all of these models have six terms. Fitting any of these three models results in improvements of RMSE by at least an order of magnitude over single- or dual-site Langmuir or Tóth. However, as the temperature increases, the RMSE decreases significantly for simpler models. As a result, the RMSE for the fitting of the ChemiPhysisorption and (single-site) Langmuir models on the CO_2_ isotherm measured on Purolite at 393 K are practically identical at 0.0025 for both despite Langmuir only using two parameters. This indicates that at higher temperatures, the adsorbate–adsorbent interaction can be well described by a single site with no heterogeneity factor needed. On the other hand, at lower temperatures, the interaction must be described through at least two sites with differing strength of interaction represented by *K*_*i*_; often, this is ascribed to the two separate processes of chemical and physical adsorption. It should also be noted that the distinction between the RMSE of TSLangmuir, DSTóth, and ChemiPhysisorption is very minimal at all temperatures. These models all have exactly six parameters; therefore, this similarity is unsurprising. Therefore, the relative utility of these models will be determined in their ability to predict Δ*h*_ads_ and then isotherms.

**Figure 2:**
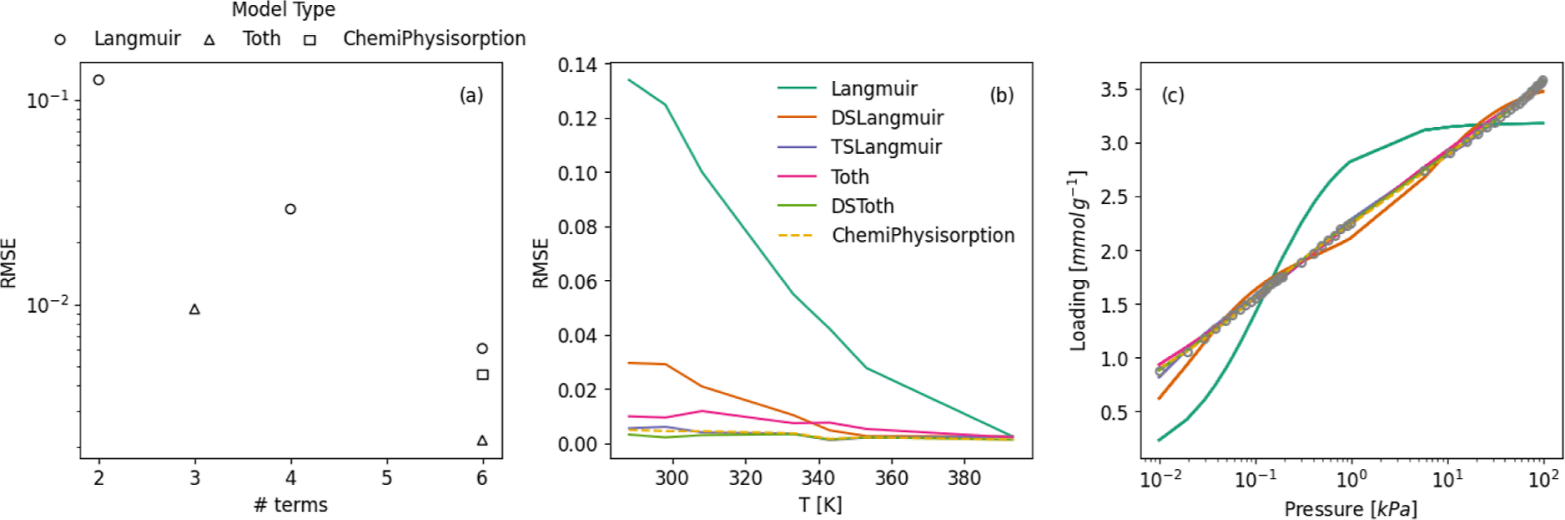
Results of fitting the Whittaker-consistent model isotherms to CO_2_ isotherms measured on Purolite.^6^ (a) shows the RMSE as a function of the number of terms for isotherms measured at 298 K—models are divided according to model type; (b) shows the RMSE as a function of temperature for each model, and (c) displays an example of the model fits for a single CO_2_ isotherm measured on Purolite at 298 K with *x*-axis in log-scale to display low-pressure fitting. All fits are attempted with maximum number of function evaluations set to 10^4^.

In order to evaluate the efficacy of using the multisite Whittaker approach, Figures 3 and S1 show the comparison of the Δ*h*_ads_ calculated using each of the Whittaker models to those calculated by Low et al. using the Clausius–Clapeyron method (eq 17) on the same experimental data. Isotherms were divided into the same subsets as used in Low et al.’s work, i.e., 333–393 K, 333–343 K, and 288–308 K. The Langmuir model (subfigures a, g, and m of Figures 3 and S1) yields a Δ*h*_ads_ that is constant as a function of loading due to the fact that eq 14 reduces to Ψ = *KP* for *i* = *t*_1_ = γ_1_ = 1. As CO_2_ is below its *P*_*t*_ (518 kPa) for the entire isotherm, Δ*H*_vap_ is taken as that at *P*_*t*_ throughout, as opposed to using enthalpy of sublimation.^22^ In addition, the deviation from ideality in this pressure range is minimal, and thus, *Z* can be assumed to be 1 for all loadings.

**Figure 3:**
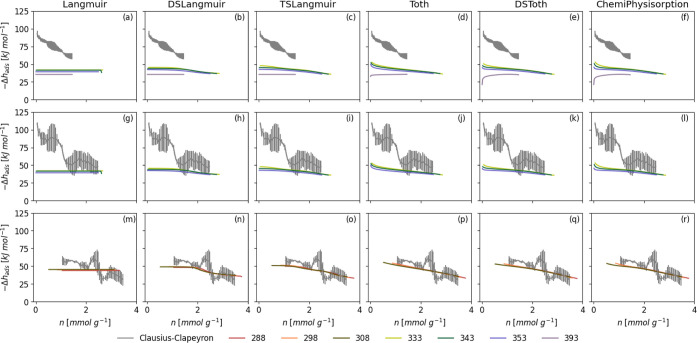
Comparison of isosteric heats of adsorption of CO_2_ on Purolite calculated using the Clausius–Clapeyron method versus the Whittaker approximation for all Whittaker-consistent models. In each row, the Whittaker approximations are taken at the same temperatures as were used for the Clausius–Clapeyron calculation, i.e., 333–393 K, 333–343 K, and 288–308 K for (a–f), (g–l), and (m–r), respectively. These are the same temperature ranges used for the calculations performed in the original work.^6^ In order to ensure that every model can be fit to the experimental isotherm, the maximum number of function evaluations was iteratively increased from 10^3^ to a maximum of 10^10^ and stopped once a fit was achieved.

For all other models, the predicted Δ*h*_ads_ varies as a function of loading due to the relative influence of multiple sites, the inclusion of a *t* exponent, or both. However, there is no real difference in the magnitude of the predicted Δ*h*_ads_ according to the model used. It should be pointed out that the predictions more closely match the Clausius–Clapeyron Δ*h*_ads_ for isotherms measured at lower temperatures. This is likely because of the reliance of the Tóth potential, eq 3 on *P*_sat_, which for isotherms measured above the adsorbate’s critical temperature (for CO_2_ 303 K) is taken as a Dubinin pseudo-saturation pressure—that is, using this method for isotherms measured at *T* > *T*_c_ relies on an additional assumption, therefore creating more uncertainty in the result. The isosteric heat of adsorption derived from fitting to the isotherm measured at 393 K unusually displays an increase as a function of loading when models with a large number of terms are used. This is probably an error as a result of overfitting and highlights the need to use the appropriate model for the isothermal system in question.

The real test is the ability of the approximate Δ*h*_ads_(*n*) to predict isotherms measured at higher or lower temperatures. Figure 4compares the predicted CO_2_ isotherms on Purolite from all models and all temperatures with the experimentally measured isotherms. An equivalent for Lewatit is given in Figure S3.

**Figure 4:**
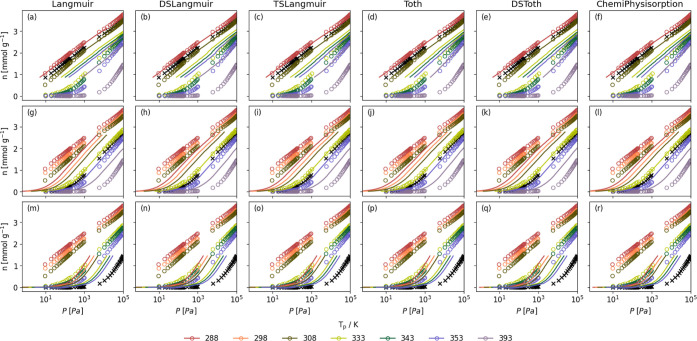
Comparison of a predicted isotherm (lines) with measured isotherms (circles, ○) for adsorption of CO_2_ on Purolite at some temperature *T*_p_. Predicted isotherms determined using the Clausius–Clapeyron equation (eq 17) with heats of adsorption derived by fitting Langmuir (a, g, m), DSLangmuir (b, h, n), TSLangmuir (c, j, o), Tóth (d, j, p), DSTóth (e, k, q), and ChemiPhysisorption (f, l, r) to an isotherm measured at *T*_e_ of 298 (a–f), 343 (g–l), and 393 (m–r). Isotherm used for modeling is marked with ×. Pressures in log scale for all isotherms to show the entire fit. This version is truncated for readability. A full version using all measured isotherms to make predictions can be found in the Supporting Information, Figure S2.

From Figures 4, S2, and S3, it is clear that regardless of the model and the original temperature, predictions are closer to the measured isotherm for smaller temperature differences, Δ*T*. This is unsurprising, as Δ*h*_ads_ is temperature-dependent, but its variation is negligible over small Δ*T*. It does not appear that the selection of the model has a significant effect on the prediction. In order to quantify how well the predicted isotherms fit the measured isotherm, the normalized RMSE between the predicted and experimental isotherms were determined in terms of pressure *P*_e_ and *P*_p_, respectively, according to eq 18

18 $$RMSE=\frac{\sqrt{\frac{\sum {\left[P_{e}\left(n\right)-P_{p}\left(n\right)\right]}^{2}}{N}}\cdot }{P_{p,\max}\left(n\right)-P_{p,\min}\left(n\right)}$$

where *N* is the number of pressure points used. The results for Purolite are shown in Figure 5a–c for all models and using a representative sample of the experimental temperatures, *T*_e_. As explained previously and shown in Figure 4, for larger values of |Δ*T*|, the RMSE gets larger. We have herein considered that a good fit is represented by an RMSE of 0.5. The point at which the RMSE crosses this line is dependent upon which model is used. In general, for prediction with |Δ*T*| less than 20 K, a reasonable prediction may be achieved, regardless of the model used. Once |Δ*T*| approaches 50 K, however, RMSEs deviate significantly, with the all models yielding RMSEs exceeding 0.5 for a Δ*T* of +50 K and a *T*_e_ of 288 K (see Figure 5a). The CO_2_ isotherm measured at 343 K on Purolite by Low et al. is approximately in the middle of the temperature range used by the authors. Thus, using this as *T*_e_ means that the effect of both a negative and positive |Δ*T*| of 50 K can be examined. As shown in Figure 5b, the RMSE stays below 0.5 for |Δ*T*| of less than 50 K, regardless of the model used. This allows us to define a reasonable limit for the usage of this method and is incorporated in the software as a warning for |Δ*T*| >50 K.

**Figure 5:**
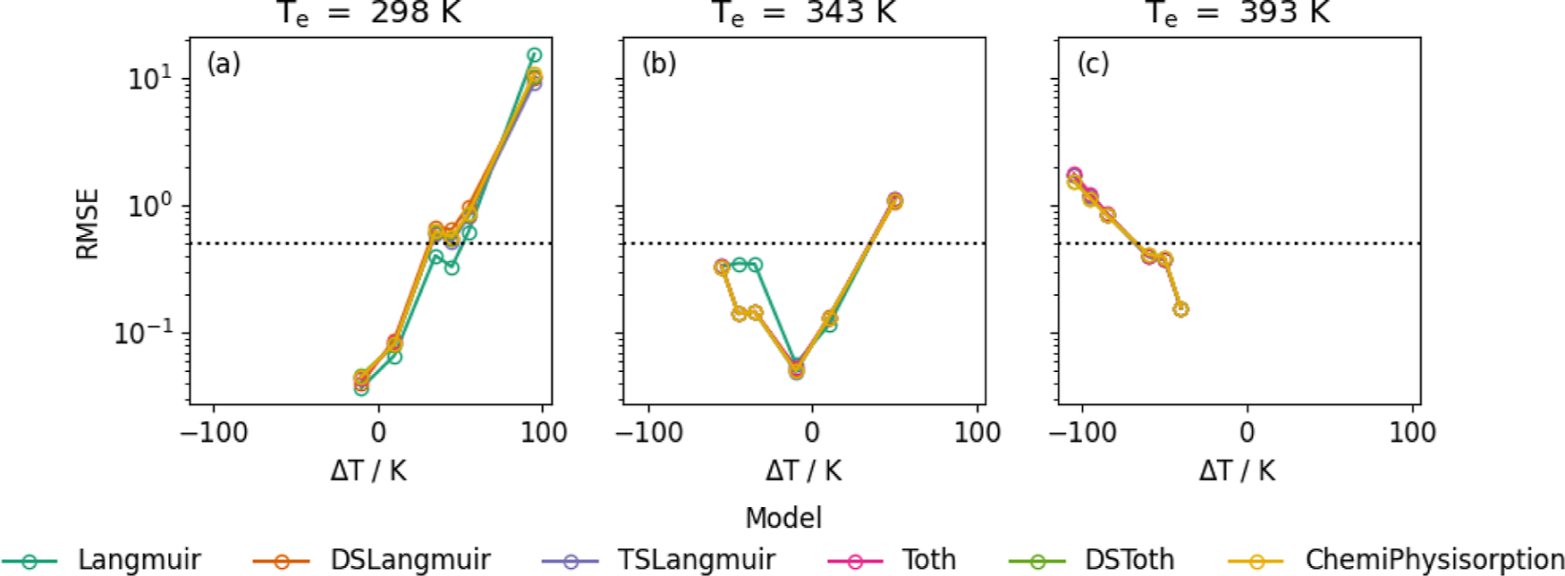
RMSE between the predicted and measured isotherms as a function of difference in temperature, Δ*T*, between the predicted and measured isotherms, calculated according to eq 18. Subfigures plotted at a selection of measured isotherm temperatures. The dotted lines indicate an RMSE of 0.5. A supplemental version of this figure with all temperatures is located at Figure S4.

This particular set of CO_2_ isotherms can be divided into three sets with narrow ranges of temperature measured at 288–308, 333–353, and 393 K. With the appropriate selection of model, one could measure only one isotherm per set and predict the remainder of the set. For example, measuring the isotherms at 298, 343, and 393 K would allow the prediction of the remaining four isotherms without resorting to the Clausius–Clapeyron method (which generates large uncertainties) and with an RMSE of no greater than 0.1 relative to the measured isotherm. This would reduce the time for measurement of CO_2_ isotherms from 14 days to 6 days, when comparing with the original method proposed by Low et al.

### Case Study Using Nitrogen Isotherms

Whittaker-derived Δ*h*_ads_(*n*) were calculated for the N_2_ isotherms on both Purolite and Lewatit using both the Whittaker method and the Clausius–Clapeyron method, as shown in supplementary Figures S6 and S7. It was necessary to manually set the lower limit for the equilibrium constants, *K*_*i*_, derived from the isotherm fitting. Due to the low loadings and thus relatively high measurement uncertainties, if there is no lower limit for *K*_*i*_, overfitting results in very small values of *K*_*i*_ on the order of 10^–9^. The method in this work is then liable to yield negative Δ*h*_ads_(*n*) values for a broad range of *n*.

The predicted isotherms for Purolite are shown in Figure 6, with the corresponding RMSE (eq 18) for all predictions in Figure 7. The respective counterparts for Lewatit are listed in Figures S8 and S9. For all models and temperatures and both adsorbents, the predictions are excellent. It appears that there is no clear advantage of using more complex models to predict isotherms, with RMSEs almost identical for most models. This is understandable as a multisite model should not be necessary to explain the mechanism of adsorption in this system. This method can be used to accurately predict isotherms with |Δ*T*| less than 20 K, with an RMSE less than 0.1.

**Figure 6:**
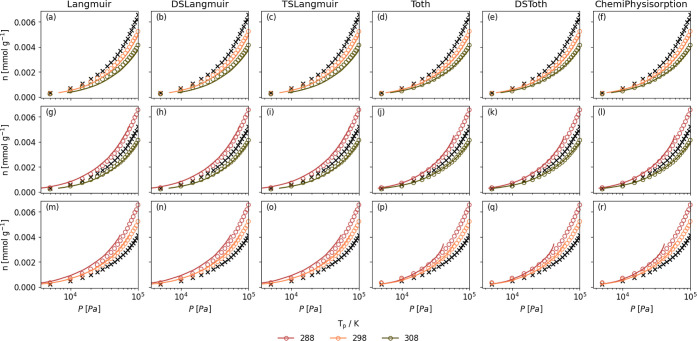
Comparison of the predicted isotherm (lines) with measured isotherms (circles, ○) for the adsorption of N_2_ on Purolite at some temperature *T*_p_. Predicted isotherms determined using the Clausius–Clapeyron equation (eq 17) with heats of adsorption derived by fitting Langmuir (a, g, m), DSLangmuir (b, h, n), TSLangmuir (c, i, o), Tóth (d, j, p), DSTóth (e, k, q), and ChemiPhysisorption (f, l, r) to an isotherm measured at a *T*_e_ of 288 (a–f), 298 (g–l), and 308 K (m–r). The isotherm used for modeling is marked with ×. Pressures in log scale for all isotherms to show the entire fit.

**Figure 7:**
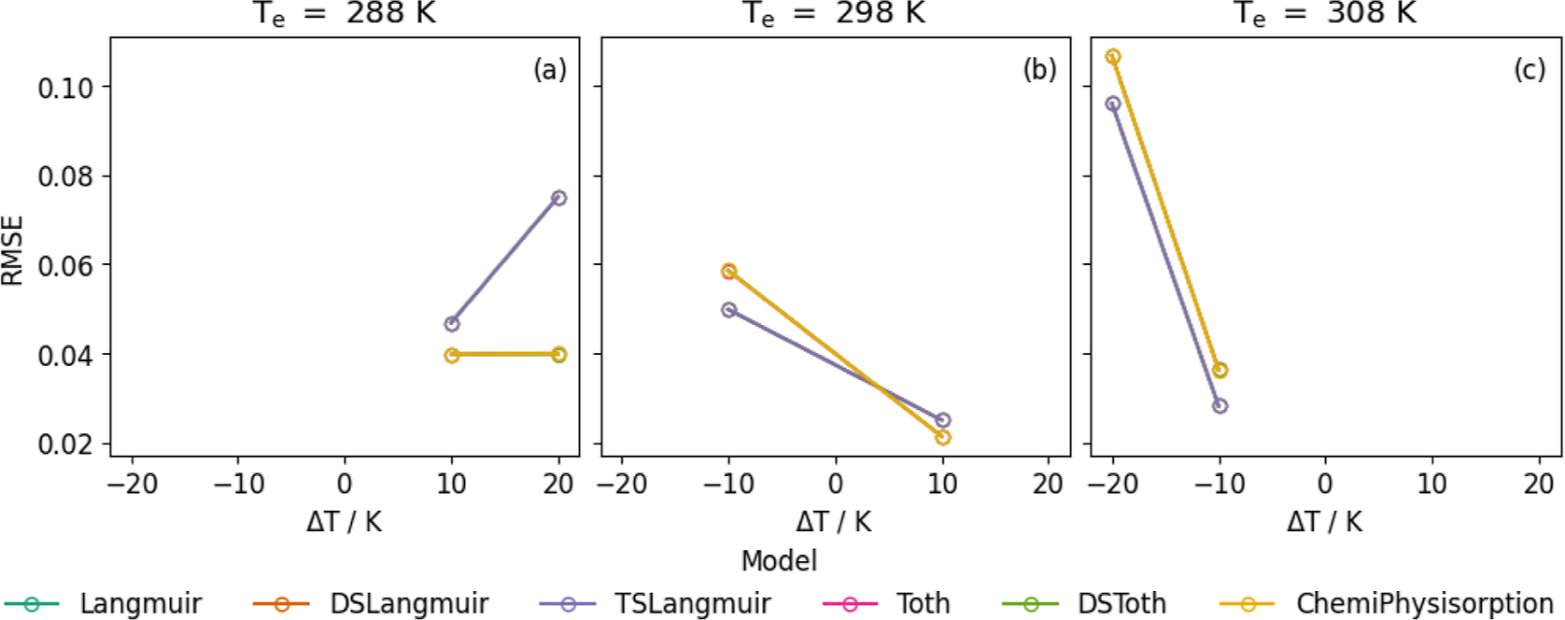
RMSE between the predicted and measured isotherms as a function of difference in temperature, Δ*T* between the predicted and measured isotherms of N_2_ on Purolite, calculated according to eq 18.

### Evaluation Based on Case Studies

The above case studies highlight that this method can have good success in evaluating the enthalpy of adsorption from a single adsorption isotherm. This can subsequently be used with a good effect to predict adsorption isotherms in a certain temperature range. There are nevertheless a number of discussion points regarding the extent of method applicability.

The approach utilized herein relies on a number of assumptions. The adsorbed phase is assumed to be liquid-like, although this is known to be ambiguous for supercritical adsorbates, micropores, and certain adsorption mechanisms. All herein method models describe adsorption in monolayers or micropores and therefore would not be applicable to systems with multilayer adsorption or pore condensation phenomena. The methods make no account or extensions for multicomponent systems. We nevertheless expect the method to be applicable to a broad range of materials and adsorbates, where the above assumptions hold.

In order to approximate Δ*h*_ads_(*n*) accurately, the fitted adsorption model must be selected not only so that it has the appropriate level of complexity for the system in question but also physical meaningful terms that describe the system.

As with other techniques relying on data fitting, the quality of the data will reflect highly on the significance and accuracy of the subsequent results. Data should be recorded in a logarithmic pressure regime. Data should also cover as much of the uptake range as possible, from 0 to the sorption plateau. Equilibration of each measurement point is also critical.

As the subsequent prediction of isotherms at different temperatures relies on an inversion of the Clausius–Clapeyron equation, its limitations apply. In particular, the assumptions that the enthalpy of adsorption does not change within the predicted range may limit the range of temperature in complex systems, for example, where chemisorption plays a significant role. Also, given the equation’s nature, the method is only able to predict pressures from loadings that have corresponding Δ*h*_ads_, effectively meaning that pressures calculated in the predicted isotherm correspond only to loadings that exist in the experimental isotherm. As a result, isotherms at temperatures significantly different from that of the experimental isotherm will have a fairly small pressure range.

While our methods facilitate faster adsorbent characterization, we encourage users to be conscious that poor data quality and/or theoretical understanding may lead to unreasonable results.

## Conclusion

The novel multisite Whittaker approximation has allowed the prediction of CO_2_ and N_2_ isotherms on two commercial styrene-divinylbenzene sorbents at temperatures 50 K above or below a measured isotherm, with a range-normalized RMSE of <0.5. This has advanced the previous work done by Whittaker et al. by (i) extending the models usable in the approximation of the heat of adsorption and (ii) using this heat of adsorption to predict isotherms at other temperatures. Accurate prediction of isotherm relies, however, on selection of the appropriate model for the adsorbate–adsorbent systems in this work. This predictive power can reduce the number of screening experiments needed for a given adsorbent, in this case reducing the screening time by over 50%.

As this method has been released as part of the open-source pyGAPS isotherm deconvolution software, it is easily usable by researchers seeking to improve rapid screening of adsorbents direct-air CO_2_ capture, as well as H_2_ storage. In addition, the feature of predicting an adsorption surface allows users to conveniently determine the optimum conditions for the achievement of a temperature–pressure swing adsorption working capacity. We hope that researchers will assist us in further expanding and improving the methods and software.

## Supplementary Material



## Notes

Applicability of Langmuir-like models, model fitting parameters, theory and software including the schemes of software usage, heat of adsorption, prediction of isotherms, and accuracy of isotherm fitting (PDF)
