# Supplementary material for: Toward Faster Adsorbent Screening via the Multisite-Whittaker Approximation
Source: Langmuir. 2025 Aug 5;41(32):21243–51. doi: 10.1021/acs.langmuir.5c01081 (PMC12369008; doi:10.1021/acs.langmuir.5c01081)
Supplement: Supplementary file 1 [file la5c01081_si_001.pdf]

Towards Faster adsorbent screening via the Whittaker approximation

L. Scott Blankenship*,† and Paul Iacomi‡

†*School of Chemistry, University Park, University of Nottingham, Nottingham NG7 2RD, UK*

‡*Surface Measurement Systems, Unit 5, Wharfside, Rosemont Road, London HA0 4PE, U.K.*

E-mail: leo.blankenship1@nottingham.ac.uk

Table S1: Overview of herein used models and their physical applicability.

**Model**	**Applicability**
Langmuir	Monolayer adsorption on homogeneous surfaces with well defined, non-interacting identical sites.
Dual-site Langmuir	Monolayer adsorption on surfaces with two distinct, non-interacting site types
Triple-site Langmuir	Monolayer adsorption on surfaces with three distinct, non-interacting site types
Toth	Heterogeneous surfaces
Dual-site Toth	Heterogeneous surfaces with two distinct site types. Of questionable utility.
ChemiPhysisorption	Systems exhibiting both chemisorption and physisorption at separate adsorption sites, one governed by a Toth, the other by Langmuir models.

Derivation of multi-site Whittaker approximation

Whittaker et al. provided a derivation of their single-site solution to the Tóth potential using either the Langmuir or Tóth equation. This work provides the Langmuir and Tóth models using the parameter K as opposed to b, where $b = \frac{1}{K^t}$ which was used in the original work. The logic remains the same.

Langmuir

The multisite Langmuir isotherm takes the form;

$$n(P) = \sum_i n_{m_i} \frac{K_i P}{1 + K_i P} \tag{S1}$$

$\frac{\mathrm{d}n}{\mathrm{d}P}$ can be calculated from the quotient rule. The inverse is thus logically $\frac{\mathrm{d}P}{\mathrm{d}n}$

$$\left(\frac{\mathrm{d}n}{\mathrm{d}P}\right)^{-1} = \frac{\mathrm{d}P}{\mathrm{d}n} = \left[\sum_i \frac{n_{m_i} K_i}{(1 + K_i P)^2}\right]^{-1} \tag{S2}$$

Therefore

$$\Psi = \left[\sum_i \frac{n_{m_i} K_i}{1 + K_i P}\right] \left[\sum_i \frac{n_{m_i} K_i}{(1 + K_i P)^2}\right]^{-1} - 1 \tag{S3}$$

Where $i = 1$, this reduces $\Psi = KP$. For more sites there is not a simple expression.

For the sake of readability equation S3 can simply be written as;

$$\Psi = \frac{n}{P} \left[\sum_i \frac{n_{m_i} K_i}{(1 + K_i P)^2}\right]^{-1} - 1 \tag{S4}$$

Tóth

The multi-site Tóth isotherm takes the form;

$$n(P) = \sum_i n_{m_i} \frac{K_i P}{\sqrt[t_i]{1 + (K_i P)^{t_i}}} \tag{S5}$$

$\frac{\mathrm{d}P}{\mathrm{d}n}$ is therefore (using the quotient rule);

$$\frac{\mathrm{d}P}{\mathrm{d}n} = \left[\sum_i \frac{\chi_i^{\frac{1}{t_i}} n_{m_i} K_i - \chi_i^{\frac{1-t_i}{t_i}} n_{m_i} K_i^{1+t_i} P^{t_i}}{\chi_i^{\frac{2}{t_i}}} \right]^{-1} \tag{S6}$$

Where $\chi_i = 1 + (K_i P)^{t_i}$

If $t_i = 1$ for all i equation S6 reduces to equation S2. Thus a rather cumbersome expression for Ψ is

$$\Psi = \left[\frac{\sum_i \frac{n_{m_i} K_i}{\sqrt[t_i]{1+(K_i P)^{t_i}}}}{\sum_i \frac{\chi_i^{\frac{1}{t_i}} n_{m_i} K_i - \chi_i^{\frac{1-t_i}{t_i}} n_{m_i} K_i^{1+t_i} P^{t_i}}{\chi_i^{\frac{2}{t_i}}}} \right] - 1 \tag{S7}$$

This can then be simplified as follows;

$$\Psi = \left[\frac{\sum_i \frac{n_{m_i} K_i}{(1+(K_i P)^{t_i})^{\frac{1}{t_i}}}}{\sum_i \frac{n_{m_i} K_i \left(1 - \frac{(K_i P)^{t_i}}{1+(K_i P)^{t_i}}\right)}{(1+(K_i P)^{t_i})^{\frac{1}{t_i}}}} \right] - 1 = \left[\frac{\sum_i \frac{n_{m_i} K_i}{(1+(K_i P)^{t_i})^{\frac{1}{t_i}}}}{\sum_i \frac{n_{m_i} K_i \left(1+(K_i P)^{t_i}\right)^{-1}}{(1+(K_i P)^{t_i})^{\frac{1}{t_i}}}} \right] - 1 \tag{S8}$$

$$\Psi = \left[\frac{\sum_i \frac{n_{m_i} K_i}{(1+(K_i P)^{t_i})^{\frac{1}{t_i}}}}{\sum_i \frac{n_{m_i} K_i}{(1+(K_i P)^{t_i})^{\frac{t_i+1}{t_i}}}} \right] - 1 \tag{S9}$$

Software Design

Modifications of the Adsorbate class

The `Adsorbate` class contains a number of functions to calculate or return adsorbate properties. The saturation pressure of an adsorbate at some temperature can be calculated using the class function `saturation_pressure()` which relies on either looking up this value from the adsorbate's `properties` dictionary or calculating it from the `CoolProp` backend. This of course returns an error if the temperature input is above the critical temperature (`t_critical()`) of the adsorbate. In order to allow for the calculation of an adsorbate's pseudo-saturation pressure,[2] a new class function, `dubinin_pseudo_saturation_pressure()` is included. The use of this is toggled by a new boolean parameter `pseudo` in `saturation_pressure()`.

Additionally, as the general heat equation and thus the multi-site Whittaker approximation requires the compressibility Z of the adsorbate at high pressures (see equations 4, 16) calculation of Z as a function of temperature and pressure was added to the `Adsorbate` class. This was implemented by creating the `compressibility()` class function, which in turn uses the `PropsSI` module of the `CoolProp` backend.

Whittaker enthalpy module

A preliminary version of the Whittaker enthalpy module, `enth_sorp_whittaker` was used in two previous publications.[3,4] This module has been present in the main pyGAPS repository as of commit 1ec9c23 in March 2023, and allowed implementation of the Whittaker approximation using the Tóth or Langmuir isotherm models (`Langmuir, DSLangmuir, TSLangmuir, Toth, DSToth, ChemiPhysisorption`). The module has now been expanded to account for all Whittaker-consistent models present in `pygaps.modelling._WHITTAKER_MODELS`. A basic example of the use of this module can be found in scheme 1. The principal function, `enthalpy_sorption_whittaker` will

run with a single input parameter, `isotherm` which should be pyGAPS `PointIsotherm` or `ModelIsotherm` object. The next two parameters, `branch` and `model` default to `'ads'` (adsorption) and `'Toth'` respectively. The `model` parameter can be set to a single model, a list of models, or `'guess'`, i.e. all `_WHITTAKER_MODELS`. In the case of using a `ModelIsotherm` its pressure unit must be in Pa, whereas when using a `PointIsotherm`, the pressure and temperature units are first converted to Pa and K respectively, then the best fit of the specified models is selected using the `pygaps.modelling.model_iso()` function. In addition, the maximum number of function evaluations `'max_nfev'` can be passed to `pygaps.modelling.model_iso()` from the keyword arguments of `enthalpy_sorption_whittaker()`, which in turn is passed to the `scipy.optimize.least_squares()` fitting function using the Trust Region Reflective fitting method.[5] This is useful in cases when models do not fit with the default settings.

The parameters n_{m_i}, K_i, and t_i from the model fit are then put into lists, to be passed to the raw function `enthalpy_sorption_whittaker_raw` function, as well as the adsorbate, the isotherm temperature, T and associated P_{sat}. The raw function then calculates ΔH_{st} for every pair of values in the supplied pressure and loading vectors according to equation 16. The uncertainty in each value of ΔH_{st} is determined from the root mean square error of the model fit and the number of terms in the model applied. Finally `enthalpy_sorption_whittaker` returns a dictionary containing the loading and ΔH_{st} vectors, the `ModelIsotherm` object, and the vector of uncertainties for each calculated ΔH_{st}. The function will optionally plot a graph of ΔH_{st} against loading if the parameter `verbose` is set `True`.

A list of all Whittaker-consistent models are accessible from `pygaps.modelling._WHITTAKER_MODELS`, and a utility function, `pygaps.modelling.is_model_whittaker()` has been added to check if a given model is Whittaker-consistent.

```
[1]: import pygaps.parsing as parse
     import pygaps.characterisation as characterise
     import pygaps.prediction as predict

     isotherm = parse.isotherm_from_aif('example.aif')
```

```
[2]: whittaker = characterise.enthalpy_sorption_whittaker(
     isotherm,
     model=['Langmuir', 'Toth', 'ChemiPhysisorption'],
     verbose=True,
     branch='ads', #
     )
```

```
Attempting to model using Langmuir.
Model Langmuir success, RMSE is 0.0697
Attempting to model using Toth.
Model Toth success, RMSE is 0.00548
Attempting to model using ChemiPhysisorption.
Model ChemiPhysisorption success, RMSE is 0.0039
Best model fit is ChemiPhysisorption.
```

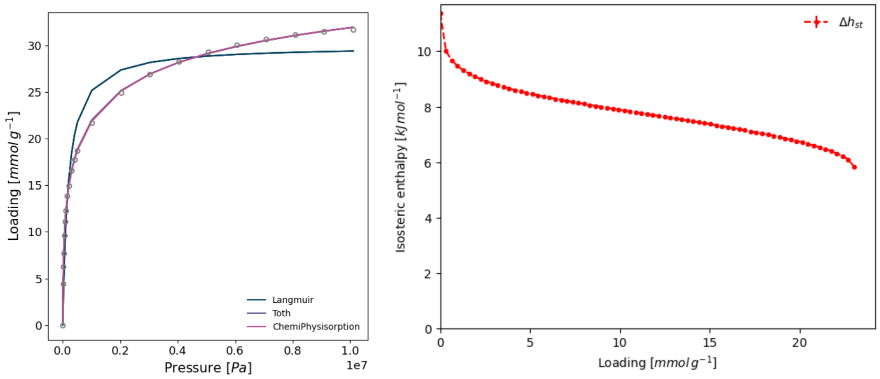

Scheme 1: Example (in jupyter notebook) of `[1]` importing an isotherm, and then `[2]` performing the Whittaker enthalpy calculation on this isotherm, and the resultant outputs. Variables in this scheme are used in later schemes.

Isotherm prediction submodule

The isotherm prediction submodule, **`enthalpy_to_isotherm`** is contained within a new **`prediction`** module, where the **`pgiast`** submodule has also been relocated. An example of the use of the submodule is given in scheme 2. Isotherm prediction is performed by calcu-

```
[3]: T_predict = isotherm.temperature+30

predict.predict_isotherm_from_enthalpy_clapeyron(
    temperature_predict=T_predict,
    isotherm=isotherm,
    isosteric_enthalpy_dictionary=whittaker,
    verbose=True
)
```

```
Using enthalpy from isosteric_enthalpy_dictionary.
```

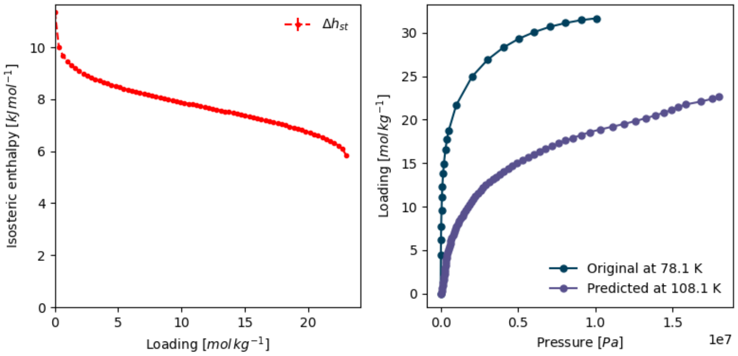

Scheme 2: Predicting a new isotherm from the original isotherm (`isotherm` from scheme 1) using; `[3]` the dictionary derived previously.

lating pressure P_p associated with a given loading n at the new temperature, T_p according to the Clausius-Clapeyron equation as in equation 17. This requires the calculation of ΔH_{st} - via the Whittaker approximation or any other means - for each point on the measured isotherm. The prediction function, `predict_isotherm_from_enthalpy_clapeyron()` at minimum requires the predicted temperature, and the `original_isotherm`. If the `original_isotherm` object does not contain isosteric enthalpy data, then a `isosteric_enthalpy_dictionary` must also be input, which can be the output of `enthalpy_sorption_whittaker()`. The function calls the bare-bones `predict_pressure_raw()` for every loading in the isotherm. As ΔH_{st} varies as a function of temperature, a warning is thrown if the difference in the experimental and predicted temperatures is more than 51 K.

Finally, an adsorption surface can be predicted using the `predict_isosurface_from_enthalpy_clapeyron()` function as shown in scheme 3.

[5]:
```
predict.predict_isosurface_from_enthalpy_clapeyron(
    original_isotherm=isotherm,
    isosteric_enthalpy_dictionary=whittaker,
    verbose=True,
    num=300,  # for smooth surface
)
```

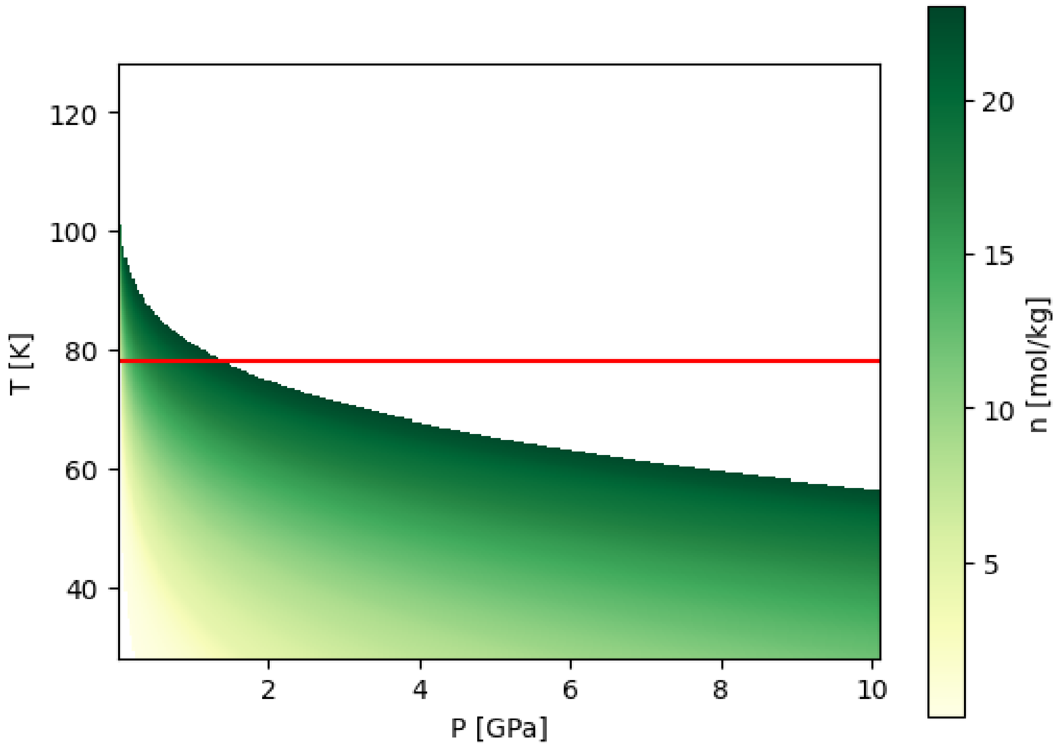

Scheme 3: An adsorption surface determined from the original `isotherm` and `Whittaker` dictionary derived in scheme 1. 300 temperatures are selected at $\pm 50K$ of the original isotherm's temperature. The red line signifies the original isotherm.

This simply calls `predict_isotherm_from_enthalpy_clapeyron()` for every value in a list of temperatures. This list is set by default to 100 temperatures in the range $T_e \pm 50K$, where T_e is the temperature of the original isotherm. The result is output as a dataframe of loadings as a function of temperature and pressure, which by default is plotted as a heatmap.

Fitting Parameters

Purolite

Table S2: Fitting information of Whittaker-consistent models to CO_2 isotherm on Purolite, measured at 288 K.

	Langmuir	DSLangmuir	TSLangmuir	Toth	DSToth	ChemiPhysisorption
RMSE	0.13	$2.96 \cdot 10^{-2}$	$5.52 \cdot 10^{-3}$	$9.94 \cdot 10^{-3}$	$3.21 \cdot 10^{-3}$	$4.93 \cdot 10^{-3}$
max_nfev	1,000	1,000	1,000	1,000	10,000	1,000
K_1	$1.05 \cdot 10^{-2}$	$1.55 \cdot 10^{-4}$	$1.99 \cdot 10^{-5}$	492.6	$1.39 \cdot 10^{9}$	$4.06 \cdot 10^{6}$
n_{m_1}	3.35	1.62	1.23	5.71	$2.03 \cdot 10^{11}$	9.76
t_1				0.15	$1.71 \cdot 10^{-2}$	$8.62 \cdot 10^{-2}$
E_a						4,036.8
K_2		$6.39 \cdot 10^{-2}$	$1.62 \cdot 10^{-3}$		4.8	$3.26 \cdot 10^{-2}$
n_{m_2}		2.14	1.2		3.45	2.74
t_2					0.26	
K_3			0.14			
n_{m_3}			1.75			

Table S3: Fitting information of Whittaker-consistent models to CO_2 isotherm on Purolite, measured at 298 K.

	Langmuir	DSLangmuir	TSLangmuir	Toth	DSToth	ChemiPhysisorption
RMSE	0.12	$2.91 \cdot 10^{-2}$	$6.08 \cdot 10^{-3}$	$9.49 \cdot 10^{-3}$	$2.17 \cdot 10^{-3}$	$4.54 \cdot 10^{-3}$
max_nfev	1,000	1,000	1,000	1,000	10,000	1,000
K_1	$8.08 \cdot 10^{-3}$	$1.4 \cdot 10^{-4}$	$1.98 \cdot 10^{-5}$	61.34	$2.29 \cdot 10^{-9}$	$4.01 \cdot 10^{6}$
n_{m_1}	3.18	1.63	1.24	5.32	5,451.3	10.98
t_1				0.17	0.16	$8.22 \cdot 10^{-2}$
E_a						4,752.65
K_2		$4.72 \cdot 10^{-2}$	$1.65 \cdot 10^{-3}$		4.36	$1.71 \cdot 10^{-2}$
n_{m_2}		1.95	1.23		4.05	3.46
t_2					0.23	
K_3			0.11			
n_{m_3}			1.51			

Table S4: Fitting information of Whittaker-consistent models to CO_2 isotherm on Purolite, measured at 308 K.

	Langmuir	DSLangmuir	TSLangmuir	Toth	DSToth	ChemiPhysisorption
RMSE	$9.99 \cdot 10^{-2}$	$2.09 \cdot 10^{-2}$	$4.03 \cdot 10^{-3}$	$1.19 \cdot 10^{-2}$	$3.01 \cdot 10^{-3}$	$4.46 \cdot 10^{-3}$
max_nfev	1,000	1,000	1,000	1,000	1,000	1,000
K_1	$5.53 \cdot 10^{-3}$	$1.1 \cdot 10^{-4}$	$1.25 \cdot 10^{-3}$	1.16	0.22	16.1
n_{m_1}	3.14	1.65	1.29	4.57	14.35	6.01
t_1				0.23	0.11	0.15
E_a						4,934.74
K_2		$2.45 \cdot 10^{-2}$	$5.23 \cdot 10^{-2}$		0.11	$2.11 \cdot 10^{-2}$
n_{m_2}		1.9	1.41		2.32	4.88
t_2					0.44	
K_3			$1.68 \cdot 10^{-5}$			
n_{m_3}			1.27			

Table S5: Fitting information of Whittaker-consistent models to CO_2 isotherm on Purolite, measured at 333 K.

	Langmuir	DSLangmuir	TSLangmuir	Toth	DSToth	ChemiPhysisorption
RMSE	$5.49 \cdot 10^{-2}$	$1.04 \cdot 10^{-2}$	$3.55 \cdot 10^{-3}$	$7.4 \cdot 10^{-3}$	$3.36 \cdot 10^{-3}$	$3.71 \cdot 10^{-3}$
max_nfev	1,000	1,000	1,000	1,000	1,000	1,000
K_1	$6.05 \cdot 10^{-4}$	$5.28 \cdot 10^{-5}$	$5.39 \cdot 10^{-4}$	$3.06 \cdot 10^{-3}$	$7.97 \cdot 10^{-6}$	$4.04 \cdot 10^{-3}$
n_{m_1}	2.68	1.74	1.45	3.89	0.78	4.45
t_1				0.37	1.22	0.26
E_a						4,146.91
K_2		$2.27 \cdot 10^{-3}$	$6.44 \cdot 10^{-3}$		$2.23 \cdot 10^{-3}$	$1.47 \cdot 10^{-3}$
n_{m_2}		1.36	0.54		2.83	3.12
t_2					0.49	
K_3			$1.73 \cdot 10^{-5}$			
n_{m_3}			1.41			

Table S6: Fitting information of Whittaker-consistent models to CO_2 isotherm on Purolite, measured at 343 K.

	Langmuir	DSLangmuir	TSLangmuir	Toth	DSToth	ChemiPhysisorption
RMSE	$4.21 \cdot 10^{-2}$	$4.77 \cdot 10^{-3}$	$1.17 \cdot 10^{-3}$	$7.57 \cdot 10^{-3}$	$1.39 \cdot 10^{-3}$	$1.64 \cdot 10^{-3}$
max_nfev	1,000	1,000	1,000	1,000	1,000	1,000
K_1	$3.71 \cdot 10^{-4}$	$2.79 \cdot 10^{-5}$	$6.64 \cdot 10^{-6}$	$9.39 \cdot 10^{-4}$	$7.08 \cdot 10^{-5}$	$4.67 \cdot 10^{-4}$
n_{m_1}	2.62	1.58	1.49	3.58	3.58	3.83
t_1				0.45	0.34	0.32
E_a						4,680.11
K_2		$9.18 \cdot 10^{-4}$	$1.71 \cdot 10^{-4}$		$9.75 \cdot 10^{-4}$	$9.21 \cdot 10^{-4}$
n_{m_2}		1.59	1.29		1.74	5.19
t_2					0.75	
K_3			$1.56 \cdot 10^{-3}$			
n_{m_3}			0.95			

Table S7: Fitting information of Whittaker-consistent models to CO_2 isotherm on Purolite, measured at 353 K.

	Langmuir	DSLangmuir	TSLangmuir	Toth	DSToth	ChemiPhysisorption
RMSE	$2.77 \cdot 10^{-2}$	$2.66 \cdot 10^{-3}$	$2.11 \cdot 10^{-3}$	$5.28 \cdot 10^{-3}$	$2.2 \cdot 10^{-3}$	$2.2 \cdot 10^{-3}$
max_nfev	1,000	1,000	1,000	1,000	1,000	1,000
K_1	$1.27 \cdot 10^{-4}$	$1.98 \cdot 10^{-5}$	$1.82 \cdot 10^{-6}$	$2.62 \cdot 10^{-4}$	$5.49 \cdot 10^{-5}$	$7.28 \cdot 10^{-5}$
n_{m_1}	2.57	1.62	2.17	3.36	2.77	2.92
t_1				0.53	0.5	0.49
E_a						5,359.56
K_2		$3.79 \cdot 10^{-4}$	$5.19 \cdot 10^{-5}$		$4.07 \cdot 10^{-4}$	$4.19 \cdot 10^{-4}$
n_{m_2}		1.44	1.22		1.17	6.25
t_2					0.94	
K_3			$4.56 \cdot 10^{-4}$			
n_{m_3}			1.16			

Table S8: Fitting information of Whittaker-consistent models to CO_2 isotherm on Purolite, measured at 393 K.

	Langmuir	DSLangmuir	TSLangmuir	Toth	DSToth	ChemiPhysisorption
RMSE	$2.54 \cdot 10^{-3}$	$2.54 \cdot 10^{-3}$	$2.54 \cdot 10^{-3}$	$2.17 \cdot 10^{-3}$	$1.34 \cdot 10^{-3}$	$1.34 \cdot 10^{-3}$
max_nfev	1,000	1,000	1,000	1,000	1,000	10,000
K_1	$1.43 \cdot 10^{-5}$	$1.43 \cdot 10^{-5}$	$1.43 \cdot 10^{-5}$	$1.48 \cdot 10^{-5}$	$1.1 \cdot 10^{-6}$	$2.26 \cdot 10^{-5}$
n_{m_1}	2.46	0.49	0.97	2.28	3.84	1.2
t_1				1.1	5.36	1.56
E_a						2.83
K_2		$1.43 \cdot 10^{-5}$	$1.43 \cdot 10^{-5}$		$2.25 \cdot 10^{-5}$	$5.22 \cdot 10^{-8}$
n_{m_2}		1.97	0.93		1.2	82.2
t_2					1.56	
K_3			$1.43 \cdot 10^{-5}$			
n_{m_3}			0.55			

Lewatit

Table S9: Fitting information of Whittaker-consistent models to CO_2 isotherm on Lewatit, measured at 288 K.

	Langmuir	DSLangmuir	TSLangmuir	Toth	DSToth	ChemiPhysisorption
RMSE	0.13	$2.82 \cdot 10^{-2}$	$6.05 \cdot 10^{-3}$	$1.05 \cdot 10^{-2}$	$3.49 \cdot 10^{-3}$	$4.68 \cdot 10^{-3}$
max_nfev	1,000	1,000	1,000	1,000	10,000	1,000
K_1	$8.34 \cdot 10^{-3}$	$1.18 \cdot 10^{-4}$	$1.54 \cdot 10^{-5}$	1,924.69	$3.03 \cdot 10^{9}$	$2.45 \cdot 10^{8}$
n_{m_1}	2.7	1.45	1.19	5.59	$4.76 \cdot 10^{5}$	14.59
t_1				0.13	$2.64 \cdot 10^{-2}$	$6.79 \cdot 10^{-2}$
E_a						4,212.8
K_2		$5.42 \cdot 10^{-2}$	$1.29 \cdot 10^{-3}$		2.61	$3.23 \cdot 10^{-2}$
n_{m_2}		1.65	1.01		2.36	2.46
t_2					0.29	
K_3			0.12			
n_{m_3}			1.34			

Table S10: Fitting information of Whittaker-consistent models to CO_2 isotherm on Lewatit, measured at 298 K.

	Langmuir	DSLangmuir	TSLangmuir	Toth	DSToth	ChemiPhysisorption
RMSE	0.12	$2.78 \cdot 10^{-2}$	$6.2 \cdot 10^{-3}$	$8.86 \cdot 10^{-3}$	$2.14 \cdot 10^{-3}$	$4.08 \cdot 10^{-3}$
max_nfev	1,000	1,000	1,000	1,000	10,000	1,000
K_1	$6.12 \cdot 10^{-3}$	$1.11 \cdot 10^{-4}$	$1.78 \cdot 10^{-5}$	132.46	$2.4 \cdot 10^{-9}$	$7.13 \cdot 10^{6}$
n_{m_1}	2.6	1.47	1.18	5.2	4,046.05	13.28
t_1				0.15	0.17	$7.46 \cdot 10^{-2}$
E_a						4,433.14
K_2		$3.9 \cdot 10^{-2}$	$1.41 \cdot 10^{-3}$		5.61	$1.6 \cdot 10^{-2}$
n_{m_2}		1.52	1.05		3.6	2.21
t_2					0.21	
K_3			0.1			
n_{m_3}			1.14			

Table S11: Fitting information of Whittaker-consistent models to CO_2 isotherm on Lewatit, measured at 308 K.

	Langmuir	DSLangmuir	TSLangmuir	Toth	DSToth	ChemiPhysisorption
RMSE	0.1	$2.11 \cdot 10^{-2}$	$3.44 \cdot 10^{-3}$	$1.05 \cdot 10^{-2}$	$2.93 \cdot 10^{-3}$	$4.57 \cdot 10^{-3}$
max_nfev	1,000	1,000	1,000	1,000	1,000	1,000
K_1	$4.2 \cdot 10^{-3}$	$9.52 \cdot 10^{-5}$	$1.64 \cdot 10^{-5}$	1.74	0.23	17.84
n_{m_1}	2.56	1.51	1.21	4.3	115.47	6.15
t_1				0.2	$7.74 \cdot 10^{-2}$	0.14
E_a						4,451.54
K_2		$2.16 \cdot 10^{-2}$	$1.17 \cdot 10^{-3}$		0.15	$1.81 \cdot 10^{-2}$
n_{m_2}		1.44	1.11		2.17	2.65
t_2					0.36	
K_3			$5.21 \cdot 10^{-2}$			
n_{m_3}			1.01			

Table S12: Fitting information of Whittaker-consistent models to CO_2 isotherm on Lewatit, measured at 333 K.

	Langmuir	DSLangmuir	TSLangmuir	Toth	DSToth	ChemiPhysisorption
RMSE	$5.96 \cdot 10^{-2}$	$9.83 \cdot 10^{-3}$	$2.61 \cdot 10^{-3}$	$1.01 \cdot 10^{-2}$	$3.38 \cdot 10^{-3}$	$3.93 \cdot 10^{-3}$
max_nfev	1,000	1,000	1,000	1,000	1,000	1,000
K_1	$6.58 \cdot 10^{-4}$	$4.59 \cdot 10^{-5}$	$7.17 \cdot 10^{-6}$	$4.59 \cdot 10^{-3}$	$1.91 \cdot 10^{-4}$	$1.54 \cdot 10^{-3}$
n_{m_1}	2.24	1.52	1.38	3.53	3.94	3.61
t_1				0.33	0.28	0.3
E_a						4,367.49
K_2		$2.5 \cdot 10^{-3}$	$2.2 \cdot 10^{-4}$		$3.06 \cdot 10^{-3}$	$3 \cdot 10^{-3}$
n_{m_2}		1.14	1.09		1.38	2.76
t_2					0.63	
K_3			$4.02 \cdot 10^{-3}$			
n_{m_3}			0.81			

Table S13: Fitting information of Whittaker-consistent models to CO_2 isotherm on Lewatit, measured at 343 K.

	Langmuir	DSLangmuir	TSLangmuir	Toth	DSToth	ChemiPhysisorption
RMSE	$4.64 \cdot 10^{-2}$	$5.11 \cdot 10^{-3}$	$1.13 \cdot 10^{-3}$	$9.19 \cdot 10^{-3}$	$2.08 \cdot 10^{-3}$	$2.31 \cdot 10^{-3}$
max_nfev	1,000	1,000	1,000	1,000	1,000	1,000
K_1	$3.92 \cdot 10^{-4}$	$2.74 \cdot 10^{-5}$	$3.42 \cdot 10^{-6}$	$1.14 \cdot 10^{-3}$	$9.23 \cdot 10^{-5}$	$3.11 \cdot 10^{-4}$
n_{m_1}	2.03	1.34	1.67	2.97	2.85	3.09
t_1				0.41	0.37	0.35
E_a						4,609.09
K_2		$1.07 \cdot 10^{-3}$	$1.23 \cdot 10^{-4}$		$1.22 \cdot 10^{-3}$	$1.23 \cdot 10^{-3}$
n_{m_2}		1.18	1		1.1	3.71
t_2					0.82	
K_3			$1.6 \cdot 10^{-3}$			
n_{m_3}			0.82			

Table S14: Fitting information of Whittaker-consistent models to CO_2 isotherm on Lewatit, measured at 353 K.

	Langmuir	DSLangmuir	TSLangmuir	Toth	DSToth	ChemiPhysisorption
RMSE	$3.4 \cdot 10^{-2}$	$3.09 \cdot 10^{-3}$	$2.11 \cdot 10^{-3}$	$7.58 \cdot 10^{-3}$	$2.37 \cdot 10^{-3}$	$2.59 \cdot 10^{-3}$
max_nfev	1,000	1,000	10,000	1,000	1,000	1,000
K_1	$1.46 \cdot 10^{-4}$	$1.87 \cdot 10^{-5}$	$4.97 \cdot 10^{-9}$	$3.57 \cdot 10^{-4}$	$8.38 \cdot 10^{-5}$	$3.99 \cdot 10^{-5}$
n_{m_1}	1.96	1.34	507.64	2.75	2.42	2.16
t_1				0.47	0.51	0.55
E_a						4,517.35
K_2		$4.86 \cdot 10^{-4}$	$4.67 \cdot 10^{-5}$		$6.17 \cdot 10^{-4}$	$5.5 \cdot 10^{-4}$
n_{m_2}		1.09	0.97		0.58	4.12
t_2					1.34	
K_3			$5.73 \cdot 10^{-4}$			
n_{m_3}			0.91			

Table S15: Fitting information of Whittaker-consistent models to CO_2 isotherm on Lewatit, measured at 393 K.

	Langmuir	DSLangmuir	TSLangmuir	Toth	DSToth	ChemiPhysisorption
RMSE	$5.08 \cdot 10^{-3}$	$5.08 \cdot 10^{-3}$	$5.08 \cdot 10^{-3}$	$4.1 \cdot 10^{-3}$	$2.1 \cdot 10^{-3}$	$2.38 \cdot 10^{-3}$
max_nfev	1,000	1,000	1,000	1,000	1,000	1,000
K_1	$9.82 \cdot 10^{-6}$	$9.82 \cdot 10^{-6}$	$9.83 \cdot 10^{-6}$	$1.13 \cdot 10^{-5}$	$7.6 \cdot 10^{-6}$	$2.44 \cdot 10^{-5}$
n_{m_1}	1.85	0.99	0.65	1.47	1.16	0.38
t_1				1.29	2.1	2.84
E_a						3,140.26
K_2		$9.82 \cdot 10^{-6}$	$9.82 \cdot 10^{-6}$		$3.17 \cdot 10^{-5}$	$1.1 \cdot 10^{-6}$
n_{m_2}		0.86	0.68		0.2	14.37
t_2					4.64	
K_3			$9.83 \cdot 10^{-6}$			
n_{m_3}			0.51			

Supplemental isotherm prediction figures

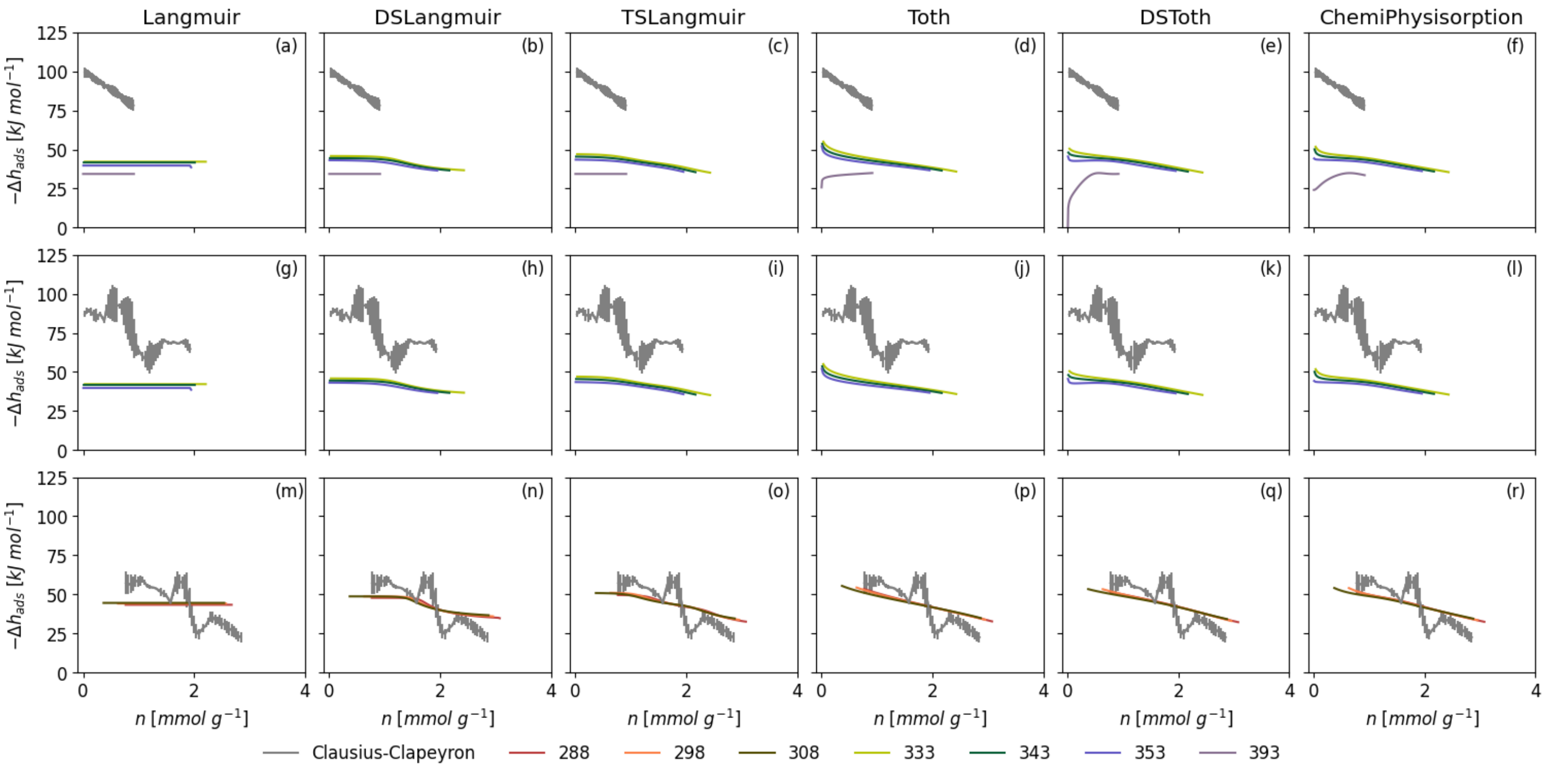

Figure S1: Comparison of isosteric heats of adsorption of CO_2 on Lewatit calculated using the Clauisus-Clapeyron method versus the Whittaker approximation for all Whittaker-consistent models. In each row, the Whittaker approximations are taken at the same temperatures as were used for the Clausius-Clapeyron calculation, i.e. 333-393 K, 333-343 K, 288-308 K for (a-f), (g-l), and (m-r) respectively. These are the same temperature ranges used for the calculations performed in the original work.[6] In order to ensure that every model can be fit to the experimental isotherm, the maximum number of function evaluations was iteratively increased from 10^3 to a maximum of 10^{10} and stopped once a fit was achieved.

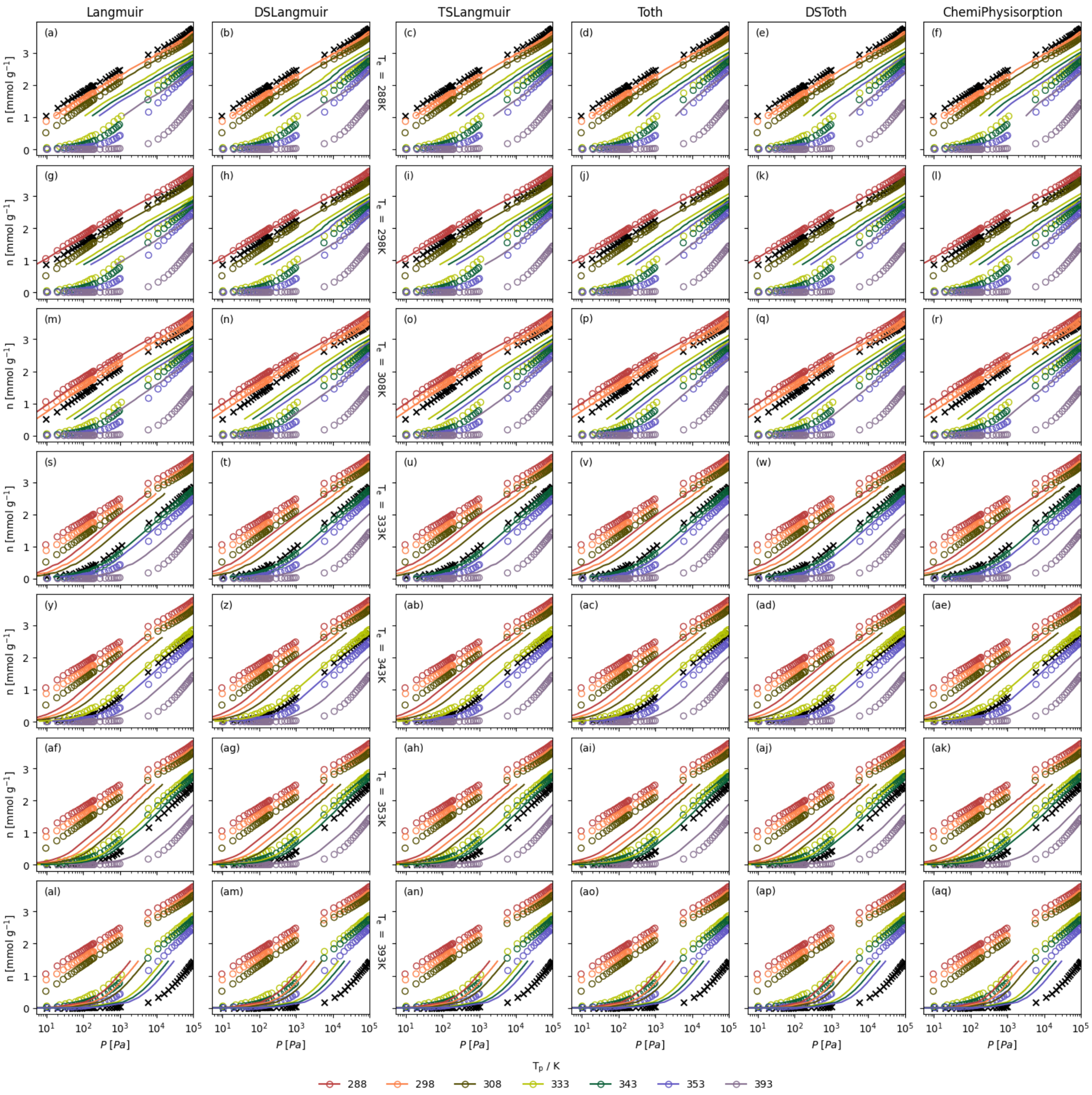

Figure S2: Comparison of predicted isotherm (lines) with measured isotherms (circles) for adsorption of CO_2 on Purolite at some temperature T_p. Predicted isotherms determined using Clausius-Clapeyron equation (equation 17) with heats of adsorption derived by fitting Langmuir (a, g, m, s, y, af, al), DSLangmuir (b, h, n, t, z ag, am), TSLangmuir (c, j, o, u, ab, ah, an), Tóth (d, j, p, v, ac, ai, ao), DSTóth (e, k, q, w, ad, aj, ap), and ChemiPhysisorption (f, l, r, x, ae, ak, aq) to an isotherm measured at T_e of 288 (a-f), 298 (g-l), 308 (m-r), 333 (s-x), 343 (y-ae), 353 (af-ak), and 393 K (al-aq). Isotherm used for modelling is marked with **x**.

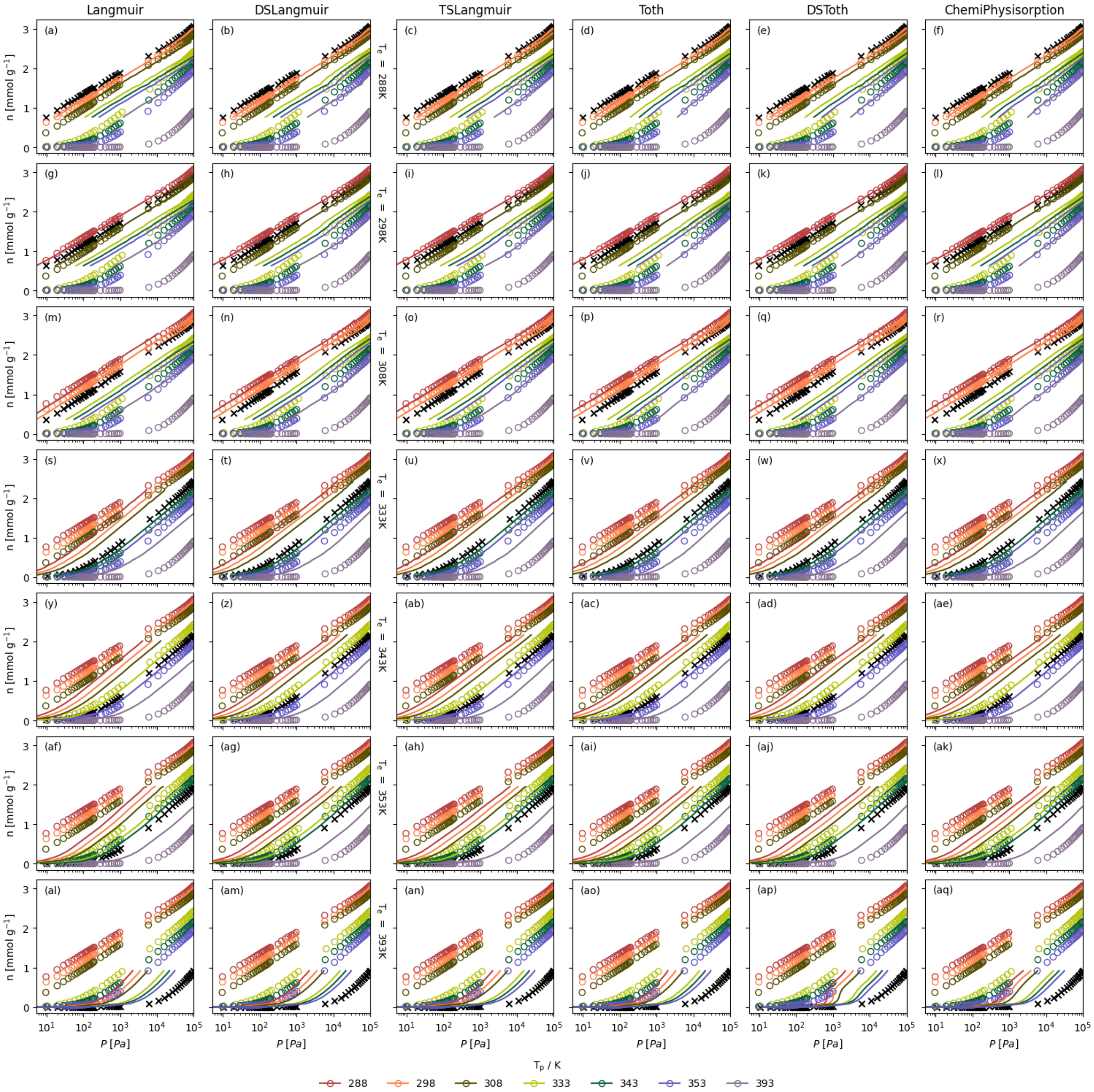

Figure S3: Comparison of predicted isotherm (lines) with measured isotherms (circles) for adsorption of CO_2 on Lewatit at some temperature T_p. Predicted isotherms determined using Clausius-Clapeyron equation (equation 17) with heats of adsorption derived by fitting Langmuir (a, g, m, s, y, af, al), DSLangmuir (b, h, n, t, z ag, am), TSLangmuir (c, j, o, u, ab, ah, an), Tóth (d, j, p, v, ac, ai, ao), DSTóth (e, k, q, w, ad, aj, ap), and ChemiPhysisorption (f, l, r, x, ae, ak, aq) to an isotherm measured at T_e of 288 (a-f), 298 (g-l), 308 (m-r), 333 (s-x), 343 (y-ae), 353 (af-ak), and 393 K (al-aq). Isotherm used for modelling is marked with **x**.

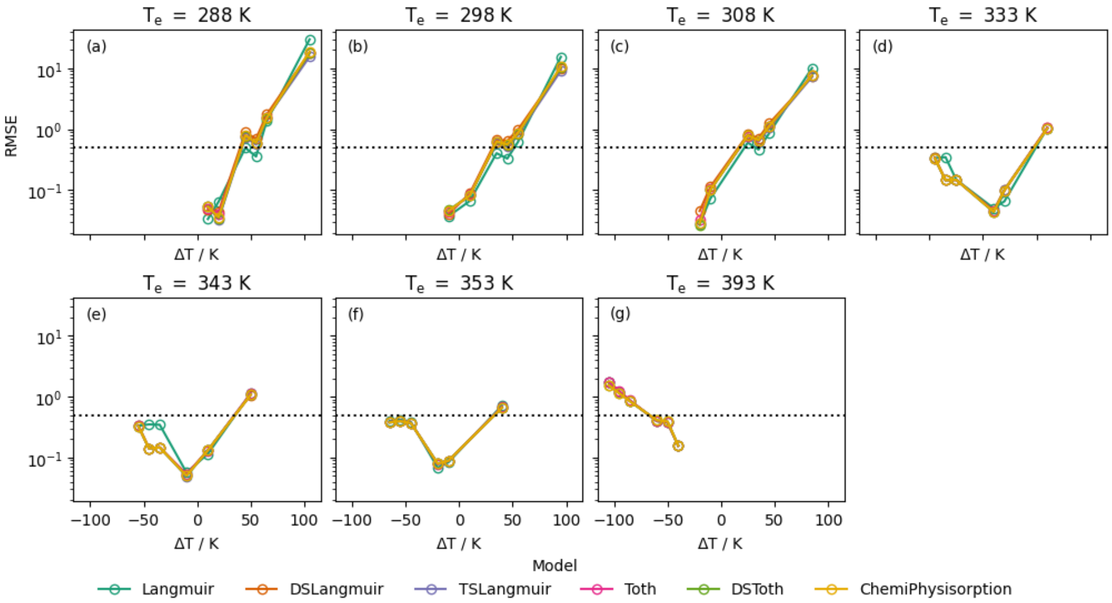

Figure S4: RMSE between the predicted and measured isotherms as a function of difference in temperature, ΔT between the predicted and measured isotherms of CO_2 on Purolite, calculated according to equation 18. All temperatures displayed.

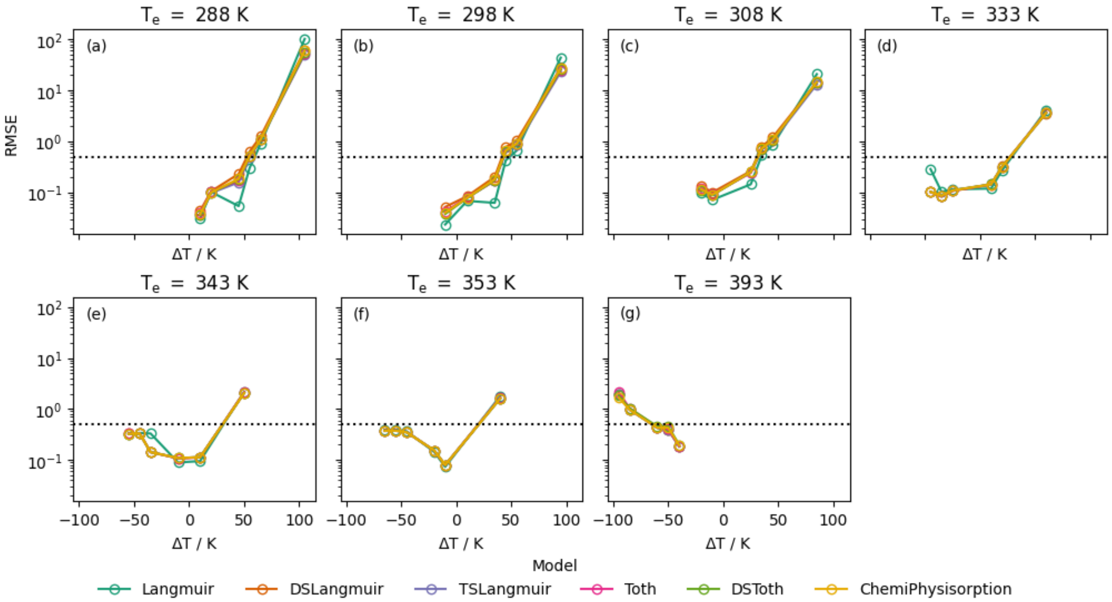

Figure S5: RMSE between the predicted and measured isotherms as a function of difference in temperature, ΔT between the predicted and measured isotherms of CO_2 on Lewatit, calculated according to equation 18. All temperatures displayed.

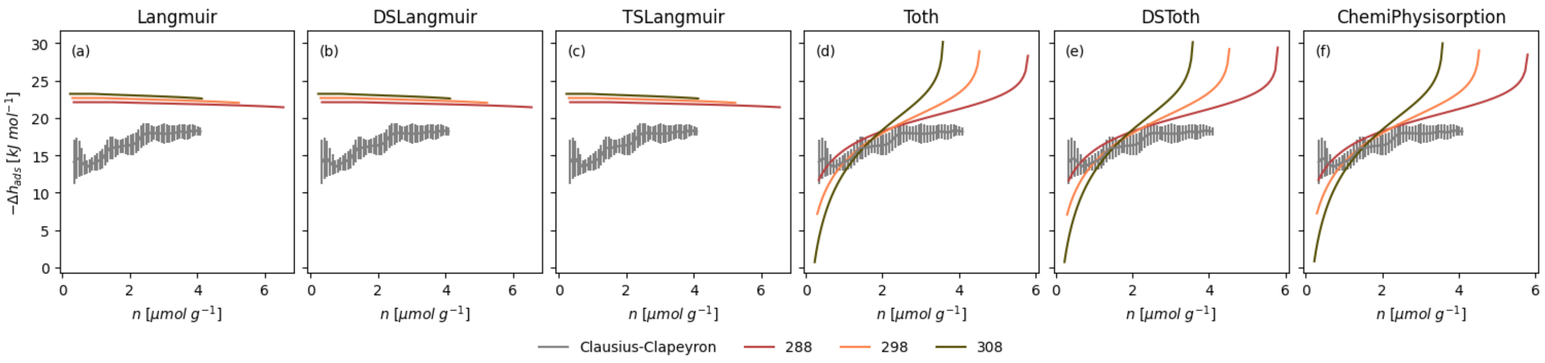

Figure S6: Comparison of isosteric heats of adsorption of N_2 on Purolite calculated using the Clauisus-Clapeyron method versus the Whittaker approximation for all Whittaker-consistent models.

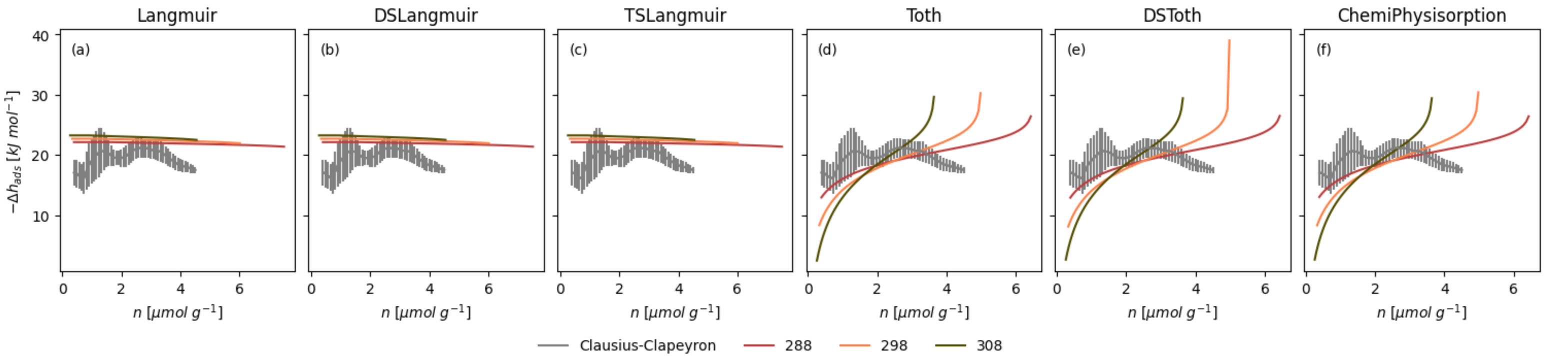

Figure S7: Comparison of isosteric heats of adsorption of N_2 on Lewatit calculated using the Clauisus-Clapeyron method versus the Whittaker approximation for all Whittaker-consistent models.

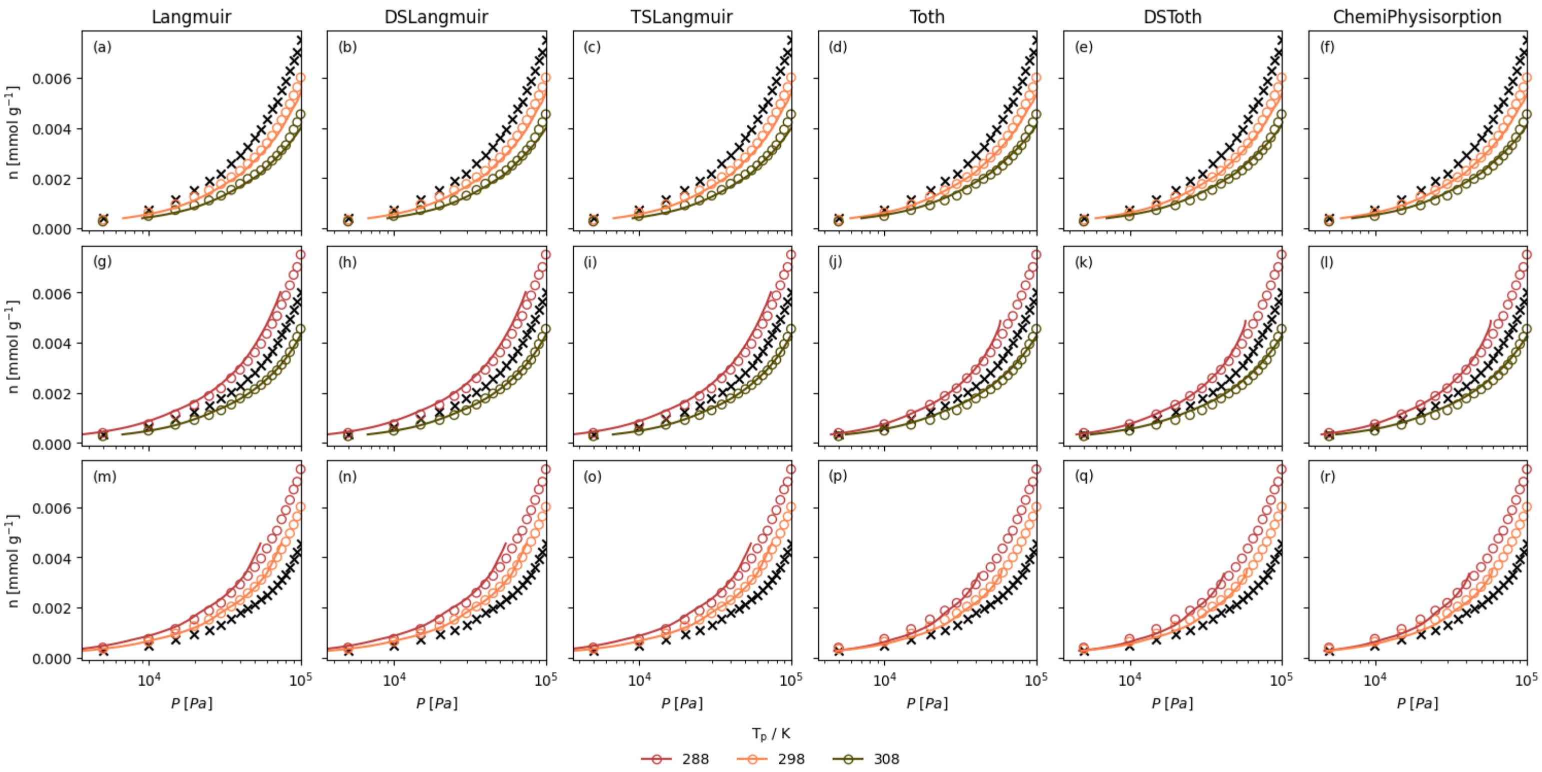

Figure S8: Comparison of predicted isotherm (lines) with measured isotherms (circles) for adsorption of N_2 on Lewatit at some temperature T_p. Predicted isotherms determined using Clausius-Clapeyron equation (equation 17) with heats of adsorption derived by fitting Langmuir (a, e, j), DSLangmuir (b, f, j), Tóth (c, g, k), and ChemiPhysisorption (d, h, l) to an isotherm measured at T_e of 288 (a-d), 298 (e-h), and 308 K (i-l). Isotherm used for modelling is marked with $\mathbf{x}$.

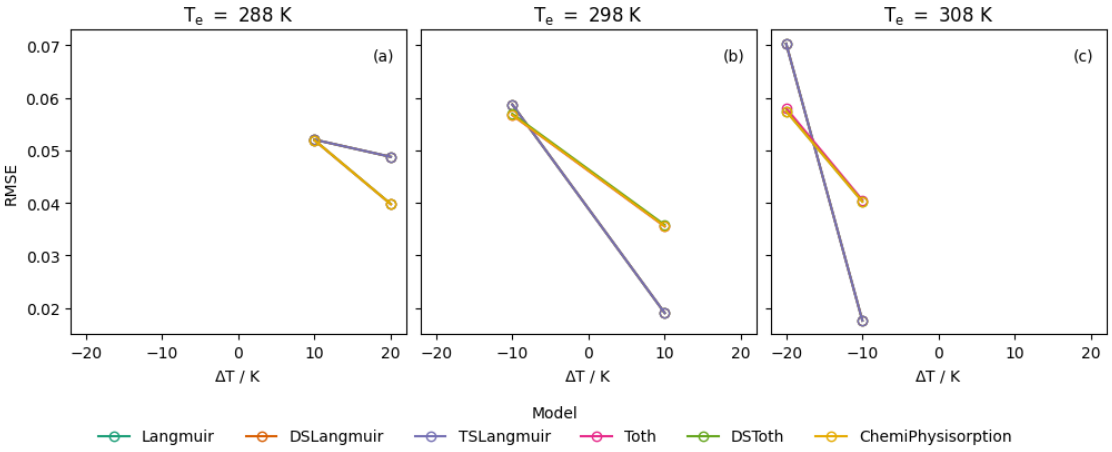

Figure S9: RMSE between the predicted and measured isotherms as a function of difference in temperature, ΔT between the predicted and measured isotherms of N_2 on Lewatit, calculated according to equation 18. Predicted isotherms are not derived from TSLangmuir as RMSE is so large as to disort the fiure. The dotted lines indicate RMSE of 0.5.

References

(1) Whittaker, P. B.; Wang, X.; Regenauer-Lieb, K.; Chua, H. T. Predicting isosteric heats for gas adsorption. *Physical Chemistry Chemical Physics* **2013**, *15*, 473–482.

(2) Dubinin, M. M. *Progress in surface and membrane science*; Elsevier, 1975; Vol. 9; pp 1–70.

(3) Alali, I.; Mokaya, R. Direct synthesis of organic salt-derived porous carbons for enhanced CO 2 and methane storage. *Journal of Materials Chemistry A* **2023**, *11*, 6952–6965.

(4) Albeladi, N.; Blankenship, L. S.; Mokaya, R. Ultra-high surface area ionic-liquid-derived carbons that meet both gravimetric and volumetric methane storage targets. *Energy & Environmental Science* **2024**, *17*, 3060–3076.

(5) (a) Virtanen, P. et al. SciPy 1.0: Fundamental Algorithms for Scientific Computing in Python. *Nature Methods* **2020**, *17*, 261–272; (b) Branch, M. A.; Coleman, T. F.; Li, Y. A subspace, interior, and conjugate gradient method for large-scale bound-constrained minimization problems. *SIAM Journal on Scientific Computing* **1999**, *21*, 1–23; (c) Byrd, R. H.; Schnabel, R. B.; Shultz, G. A. Approximate solution of the trust region problem by minimization over two-dimensional subspaces. *Mathematical programming* **1988**, *40*, 247–263.

(6) Low, M.-Y. A.; Danaci, D.; Azzan, H.; Woodward, R. T.; Petit, C. Measurement of Physicochemical Properties and CO2, N2, Ar, O2, and H2O Unary Adsorption Isotherms of Purolite A110 and Lewatit VP OC 1065 for Application in Direct Air Capture. *Journal of Chemical & Engineering Data* **2023**, *68*, 3499–3511.
